# FunSeq2: a framework for prioritizing noncoding regulatory variants in cancer

**DOI:** 10.1186/s13059-014-0480-5

**Published:** 2014-10-02

**Authors:** Yao Fu, Zhu Liu, Shaoke Lou, Jason Bedford, Xinmeng Jasmine Mu, Kevin Y Yip, Ekta Khurana, Mark Gerstein

**Affiliations:** Program in Computational Biology and Bioinformatics, Yale University, New Haven, CT 06520 USA; School of Life Science, Fudan University, Shanghai, 200433 People’s Republic of China; Department of Computer Science and Engineering, The Chinese University of Hong Kong, Shatin, New Territories Hong Kong; Broad Institute of Harvard and MIT, Cambridge, MA 02142 USA; Molecular Biophysics and Biochemistry Department, Yale University, New Haven, CT 06520 USA; Present address: Department of Physiology and Biophysics, Weill Cornell Medical College, New York, NY USA; Department of Computer Science, Yale University, New Haven, CT 06520 USA

## Abstract

Identification of noncoding drivers from thousands of somatic alterations in a typical tumor is a difficult and unsolved problem. We report a computational framework, FunSeq2, to annotate and prioritize these mutations. The framework combines an adjustable data context integrating large-scale genomics and cancer resources with a streamlined variant-prioritization pipeline. The pipeline has a weighted scoring system combining: inter- and intra-species conservation; loss- and gain-of-function events for transcription-factor binding; enhancer-gene linkages and network centrality; and per-element recurrence across samples. We further highlight putative drivers with information specific to a particular sample, such as differential expression. FunSeq2 is available from funseq2.gersteinlab.org.

**Electronic supplementary material:**

The online version of this article (doi:10.1186/s13059-014-0480-5) contains supplementary material, which is available to authorized users.

## Background

Cancer genome sequencing generally identifies thousands of somatic alterations in individual tumor genomes. A few of them, called drivers, contribute to oncogenesis, whereas the majority are passenger mutations accumulated during cancer progression [1]. Systematic studies of human cancer genomes have discovered a wide range of driver genes [2–6]. However, comparatively less effort has been invested in the noncoding portions of the genome. Recent discovery of somatic mutations in telomerase reverse transcriptase (*TERT*) promoter shows that regulatory variants may constitute driver events [7–10]. With the decrease of sequencing cost, international cancer consortia, such as TCGA (The Cancer Genome Atlas) and ICGC (The International Cancer Genome Consortium), plan to perform large-scale cancer whole-genome sequencing in the near future. Thus, there is a great demand for high-throughput computational methods to analyze those variants.

In contrast to coding variants, the functional impact of noncoding variants is more difficult to evaluate, due to the lack of knowledge about noncoding regions. The important role of regulatory variants in various diseases has generated a great deal of interest in studying noncoding sequences [11–14]. Projects aiming to uncover potential regulatory sequences, such as The Encyclopedia of DNA Elements (ENCODE) [15] and 29 Mammals Project [16], provide an unprecedented opportunity to interpret noncoding variants. Studies have shown that disease-associated single nucleotide polymorphisms (SNPs) identified by Genome-wide Association Studies (GWAS) are significantly enriched in ENCODE regions [17]. A number of tools have been developed using these data to annotate potential regulatory variants or to suggest the most likely causal variants in linkage disequilibrium with GWAS SNPs, such as Haploreg [18], RegulomeDB [19], ANNOVAR [20], GEMINI [21], FunciSNP [22], and VEP [23]. Recently, two computational approaches - GWAVA and CADD - were published to predict the deleterious effect of variants genome-wide [24,25]. These two methods utilized machine-learning models trained on potential pathogenic variants or nearly fixed/fixed human derived alleles to distinguish deleterious variants from neutral ones.

While much work has been done for germline variants, this is not the case for cancer somatic mutations. Through analyzing the variation patterns of natural polymorphisms, we have published a prototype approach (FunSeq) to identify potential noncoding drivers [26]. Here, we report a more elaborate and flexible framework - FunSeq2 - to annotate and prioritize somatic alterations integrating various resources from genomic and cancer studies. The framework consists of two components: (1) data context from uniformly processing large-scale datasets; and (2) a high-throughput variant prioritization pipeline. The data context can be rebuilt using newly available dataset. Key features of our pipeline include: (1) integrating various functional annotations to identify potential regulatory variants; (2) predicting nucleotide-level loss- and gain-of-function events; (3) examining whether variants occur in noncoding regions that are less likely to tolerate mutations, by analyzing both evolutionary and human population-level conservation; (4) systematically linking variants with target genes using data from the Roadmap Epigenomics Project; (5) incorporating network topology analysis, gene functions, and user annotations to investigate these variant-gene linkages; and (6) identifying recurrent elements from both user-input and publicly available cancer datasets. To prioritize ‘high-impact’ variants, we developed a weighted scoring scheme that takes into account the relative importance of various features, based on the mutation patterns observed in natural polymorphisms.

Besides mutations in the *TERT* promoter, no other regulatory variants have been functionally characterized as cancer drivers. Thus, due to the lack of a gold standard for regulatory cancer drivers, we used recurrent somatic mutations and known germline pathogenic variants to evaluate the performance of our method. Our method has good prediction power for both recurrent somatic and germline pathogenic regulatory variants, and more importantly it contains multiple cancer-specific features, such as differential gene expression detection between tumor and matched normal samples. As a test case, we also applied our method to an individual tumor genome with a known *TERT* promoter mutation. Our method is able to prioritize the variant and provides a hypothesis for its potential functional impact. This shows that our method can help researchers and clinicians to prioritize a few somatic regulatory mutations for further studies.

## Results and discussion

**Figure 1:**
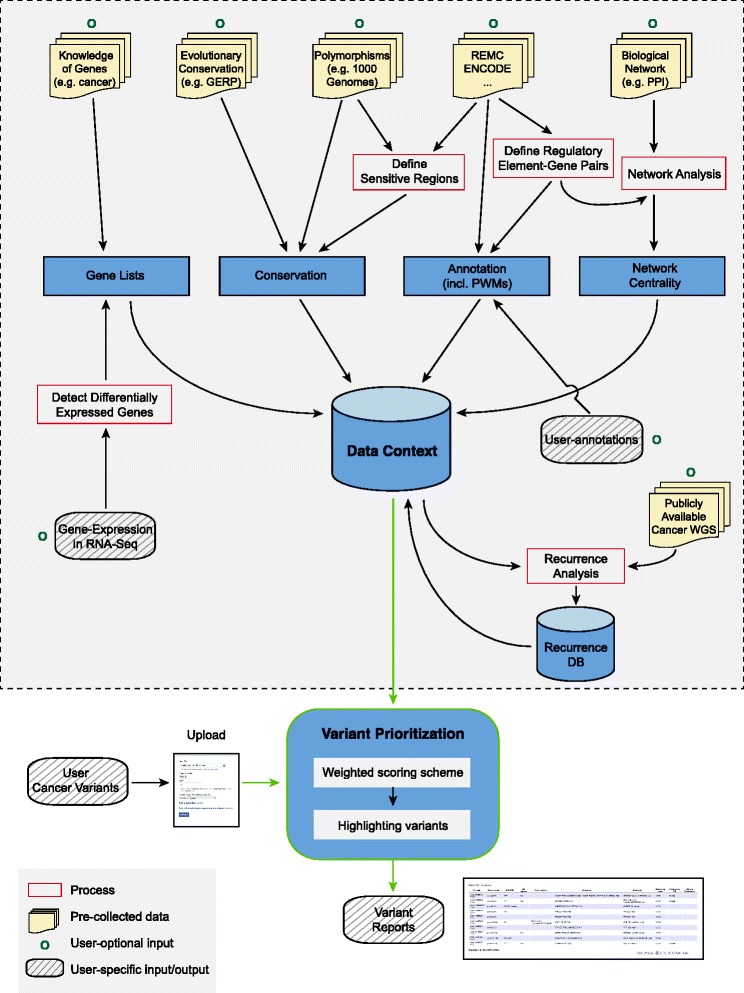
**Schematic workflow.** FunSeq2 consists of two components: creation of data context and variant prioritization. We processed large-scale genomics (such as 1000 Genomes and ENCODE data) and cancer resources to create the small-scale informative data context, as shown within the dashed rectangle. The variant prioritization pipeline will take user-input cancer variants and then annotate and score them against the data context. All features are used to annotate variants (shown in Additional file 1: Table S2), whereas a fraction of them are used to score variants (Additional file 1: Table S3) with the weighted scoring scheme. ‘Process’ contains scripts to analyze data, which can be downloaded from our website. Green arrows show the input and output of the prioritization pipeline (matched with Figure 2).

**Figure 2:**
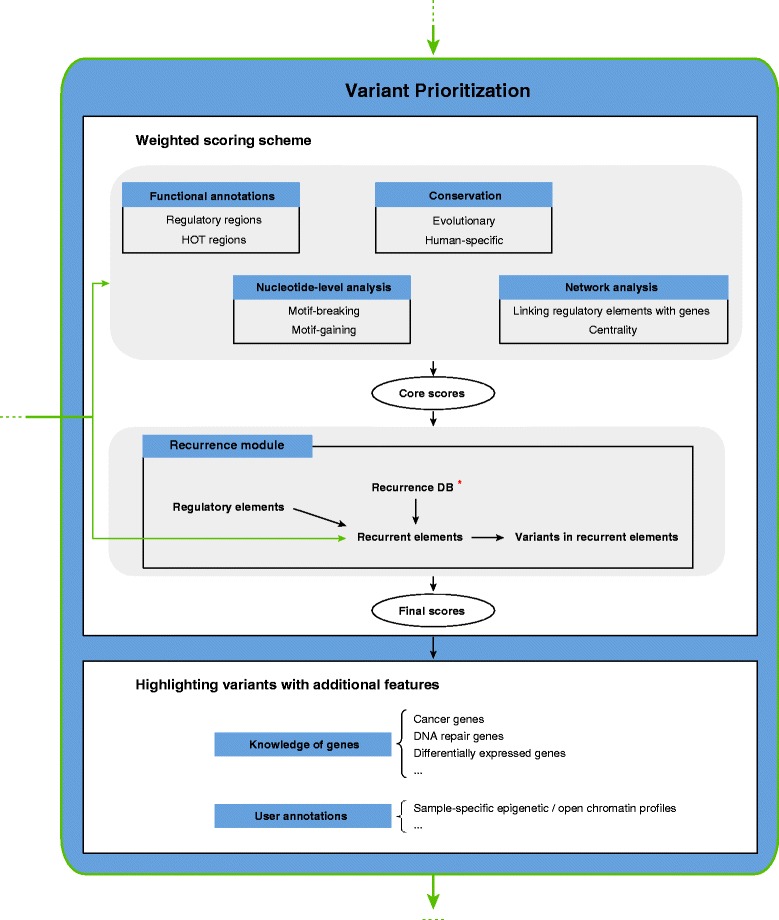
**Variant prioritization.** The variant prioritization step will annotate input variants and then score them using the weighted scoring scheme. Features used in the weighted scoring scheme can be classified into ‘functional annotations’, ‘conservation’, ‘nucleotide-level analysis’, ‘network analysis’, and ‘recurrence’. ‘Recurrence’ feature could be detected from user-input cancer samples and also from ‘Recurrence DB’ (* means optional. User can choose to use the ‘Recurrence DB’ or not). Different from other features, ‘recurrence’ depends on the user-input (for example, if user only uploads one sample and chooses not to use the ‘Recurrence DB’, then ‘recurrence’ feature will not be observed for any variant). Each feature is assigned a weighted score (Material and methods). Scores obtained from the top grey panel are called ‘core scores’, which is independent of the user’s choice (see above for ‘recurrence’ feature). Variants with the ‘recurrence’ feature are assigned an additional score in the final output. In addition to features used in the scoring scheme, other features are used to highlight potentially interesting variants, such as variants associated with known cancer genes.

High-throughput technologies have generated huge amount of genomics data in the past few decades. How to mine and integrate these data to tackle particular scientific question remains a challenge. In this study, we first build an organized data context by processing large-scale genomics and cancer resources into small-scale informative data and then use them to annotate and prioritize cancer somatic alterations. The workflow is depicted in Figure 1 and the detailed description of variant prioritization is in Figure 2.

### Variants in potential regulatory elements

Regulatory elements, especially promoters and enhancers, are capable of regulating the expression of specific genes. We collected functional annotations from ENCODE [15] (transcription factor binding sites and the high-resolution motifs within them, enhancers from genome segmentations and DNase I hypersensitive sites) and regions that are highly occupied by transcription factors (HOT) from *Yip et al.* [27] to annotate variants in potential regulatory sequences. We evaluated their functional effect from sequence and network levels in the following sections.

### Nucleotide-level impact of regulatory variants

Regulatory mutations can cause transcriptional alterations by either loss-of or gain-of- function effects. Loss-of-function events in transcription factor binding motifs are likely to cause deleterious impact [26,28,29]. Variants decreasing the position weight matrix (PWM) scores could potentially alter the binding strength of transcription factors, or even eliminate the binding. Our framework consists of a module to detect motif-breaking events - defined as variants decreasing PWMs (Material and methods). Meanwhile, gain of new binding sites caused by somatic mutations can constitute driver events [7–10]. To the best of our knowledge, there is no automated tool to detect such events in whole tumor genomes. We incorporated a gain-of-motif scheme to scan and statistically evaluate [30] all possible motifs created by mutated alleles in promoter or enhancer regions. For each variant (SNV or indel), we concatenated it with +/- 29 bp nucleotides around it and calculated sequence scores for each possible motif against the PWMs. Gain-of-motif events are identified when the sequence score with mutated allele is significantly higher than the background (*P* <4e-8), whereas that with germline allele is not. As discussed later, our scheme is validated by the detection of motif-gaining events caused by the two driver mutations in *TERT* promoter (Additional file 1: Table S1).

### Variants in conserved regions

Sequences that are under strong negative or purifying selection are thought to have important biological functions [31]. In previous studies, oncogenes or tumor suppressor genes are found to experience higher intensity of selective pressure than other disease-related and non-disease genes [32]. Cross-species genome comparison is a powerful approach to identify evolutionary conserved sequences. For example, GERP [33] is developed to estimate the position-specific evolutionary constraint; sequences that are shared across species are defined as ultra-conserved elements [31]. Meanwhile, human population-level constrained regions are identified from 1000 Genomes [26,34] using depletion of common polymorphisms. We combined these data to detect potential deleterious variants in noncoding sequences. Each variant will be annotated with its corresponding conservation information.

### Linking regulatory elements with likely target genes

Functional genomic studies have characterized biological functions of a large number of genes. Linking regulatory variants with coding genes, especially those well-known cancer driver genes, will help us understand the regulatory mechanisms that govern oncogenesis and potentially benefit therapeutic treatment. Positioned distant to their target genes, regulatory elements regulate gene expression through long-range interactions [35]. The linkages between regulatory elements and genes remain elusive. To explore likely functional consequences of regulatory variants, we comprehensively define regulatory element-target gene pairs through correlating various epigenetic modifications with gene expression levels. We consider the enhancer marks H3K4me1 and H3K27ac as two types of activity signals, and DNA methylation as an inactivity signal. Using ChIP-Seq and RNA-Seq data from the Roadmap Epigenomics Mapping Consortium (REMC), for each regulatory element-candidate target gene pair, we computed the correlations of H3K4me1 and H3K27ac and the anti-correlations of DNA methylation at the regulatory element with gene expression levels across 20 tissue types (Material and methods). In total, we identified approximately 2,448 K significant associations involving roughly 1,279 K regulatory elements and 17 K genes (Material and methods; Additional file 1: Figure S1). All noncoding variants in these regulatory elements can be associated with potential target genes (with various association confidences). To incorporate the ever-increasing amounts of genomic data, we include a pipeline for users to extend the data context with their own data. For example, users can input annotation regions or chromatin marks to find novel associations between regulatory elements and coding genes (Additional file 1).

### Network analysis of variants associated with genes

Unlike germline mutations, somatic alterations are not expected to be under organism-level evolutionary selection pressure and thus are more likely to affect functional centers in gene interaction networks [36]. Network studies have found that cancer genes possess high topological centralities, even higher than essential genes [26,36]. We used the regulatory element-target gene pairs to connect noncoding variants to a variety of networks: protein-protein interaction, regulatory and phosphorylation networks [26,35,37]. For each noncoding variant, we calculated the scaled network centrality (the percentile after ordering centralities of all genes in a particular network) of the associated gene in each network (Material and methods). Among the different network centralities, we used the maximum centrality as the network disruptive measure of the variant. The higher this value, the more likely the variant is to be deleterious. We make the scheme flexible so it can integrate user networks in addition to the pre-collected networks.

### Recurrent elements across cancer samples

One criterion to identify cancer driver genes is to examine their mutational recurrence across multiple samples [3]. We extended the concept to noncoding regulatory elements, such as transcription factor binding sites. Our method is able to detect sites, genes, and regulatory elements that are mutated in at least two samples from user-input (Material and methods).

When having small sample sizes, comparisons with available tumor genomes would be useful to discover recurrent mutations. Related to this, we have created a recurrence database (Recurrence DB) (including regulatory elements, coding genes and the same-site mutations) with publicly available cancer whole-genome sequencing data. Currently, we have identified recurrent loci or sites from 570 samples of 10 tumor types [38–40] and from COSMIC [41] (Table 1; Material and methods). Variants in user-input tumor genomes are compared to the recurrence database and the results in different cancer types are reported in the output. The use of the database along with our framework would provide higher confidence in prioritization of regulatory drivers (Figure 2). The database will be updated with newly available dataset.

**Table 1:** **Summary of recurrence database**

**Cancer type**	**Samples (n)**	**Somatic mutations (SNVs) (n)**	**Recurrent elements | Genes | Mutations (n)**
AML	7	271-1,068	1
Breast	119	1,043-67,347	69,140
CLL	28	522-3,338	709
Liver	88	1,348-25,131	74,144
Lung adeno	24	9,284-297,569	162,165
Lymphoma B cell	24	1,502-37,848	4,233
Medulloblastoma	100	44-47,440	2,793
Pancreas	15	1,096-14,998	2,591
Pilocytic astrocytoma	101	2-926	58
Prostate	64	1,430-18,225	36,327
COSMIC recurrent regulatory mutations	-	-	10,041

### Weighted scoring scheme to prioritize variants

**Figure 3:**
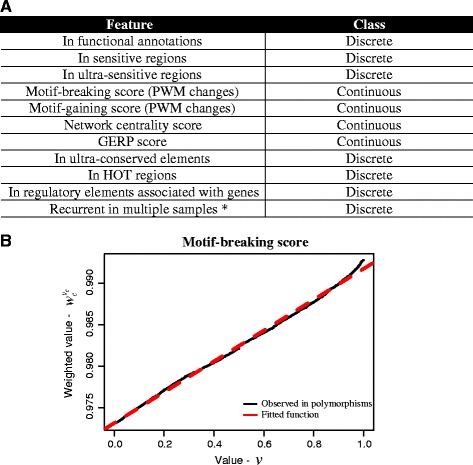
**Weighted scoring scheme. (A)** Features used in the weighted scoring scheme. Features can be classified into two classes: discrete and continuous. Discrete features are binary, such as in ultra-conserved elements or not. For continuous features, taking the ‘motif-breaking score’ as an example, the values would be the changes in PWMs. * - the feature depends on the user (see Figure 2); **(B)** We weighted each feature based on the mutation patterns observed in natural polymorphisms. Features that are frequently observed are less likely to contribute to the deleteriousness of variants and are weighted less (entropy-based method, details described in Material and methods). For a continuous feature, such as the ‘motif-breaking scores’, we calculated a weight for each observed value. The x-axis is the observed motif-breaking score and the y-axis is the corresponding weight. The black line shows the values observed in natural polymorphisms. We then fit a smooth curve (the red dashed line) to obtain continuous weights for all possible motif-breaking scores.

All of the above features are used to annotate and score variants (Figure 3). To integrate the various features to predict ‘high-impact’ somatic alterations, we developed a weighted scoring scheme, taking into account the relative importance of each feature (Figure 2). In general, features can be classified into two classes: discrete and continuous. Discrete features are binary, such as in ultra-conserved elements or not. For continuous features, taking the ‘motif-breaking score’ as an example, the values would be the changes in PWMs. We weighted each feature based on the mutation patterns observed in natural polymorphisms (Material and methods; Additional file 1: Table S3). Constrained by selective pressure, natural variations tend to arise in functionally unimportant regions. Thus, features that are frequently observed are less likely to contribute to the deleteriousness of variants and are weighted less. We calculated the information content to denote the importance of each feature. For each cancer mutation, we scored it by summing the information contents of all its features (details in Material and methods). Variants ranked on top of the output are those with higher scores and are most likely to be deleterious.

### Highlighting variants using prior knowledge of genes and user annotations

Interpretation of the functional impact of noncoding variants can be greatly enhanced if the function of its target protein-coding gene is known. Many cancer genes are known to play a crucial role in cell proliferation and DNA repair. We integrated prior knowledge of genes, such as known cancer-driver genes [2,42], genes involved in DNA repair [43] and actionable genes (‘druggable’ genes) [44] to highlight noncoding variants that are likely to be involved in cancer development and growth or their associated genes could be used as drug targets. In addition, user-specific gene lists can be easily input (Figure 1 and Additional file 1).

Variants in regulatory elements may disrupt the expression of coding genes. Differential expression of target genes in cancer samples indicates the potential effect of noncoding variants. We provide a ‘differential gene expression analysis’ module (Figure 1 and Material and methods) to detect differentially expressed genes in cancer samples (relative to matched normal) from RNA-Seq data. Lists of differentially expressed genes can be generated and used to annotate variants.

We also provide an option for users to incorporate their own annotations. Impact of variants in regulatory regions is generally restricted to *cis*-acting effects that control the spatial and temporal patterns of gene expression [13]. Activation of regulatory elements is associated with the underlying epigenetic or open chromatin landscape, which is largely cell-line specific [45]. For example, enrichment of H3K27ac may indicate an active state of enhancers in a particular sample. Therefore, it would be useful to incorporate sample-specific epigenetic or open chromatin profiles, if available, to highlight variants in activated or inactivated regulatory sequences.

All features used in our method and corresponding details are described in Additional file 1: Table S2.

### Performance on regulatory cancer somatic variants and germline pathogenic variants

#### Recurrent somatic variants

**Figure 4:**
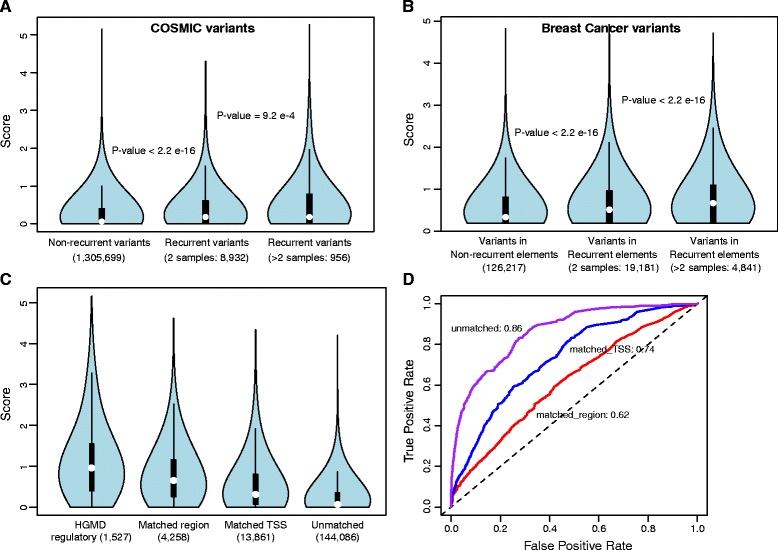
**Application to pathogenic germline and cancer somatic regulatory variants. (A)** Score distribution of variants based on their recurrence in COSMIC. Variants are classified into three categories based on the number of recurrent samples; **(B)** Score distribution of variants based on recurrent regulatory elements in 119 breast cancer samples. Recurrent regulatory elements are defined as elements mutated in more than one sample, such as the same DNase I hypersensitive site; **(C)** Prediction scores for regulatory variants in HGMD and controls; **(D)** ROC curves comparing HGMD variants with controls. All scores in this figure are ‘core scores’ (Figure 2).

Currently, only two known regulatory variants are thought to act as cancer drivers. Hence, to evaluate the performance of our scoring scheme, we used recurrence to approximate the deleteriousness of somatic variants. Recurrence is considered as one potential sign of positive selection among tumors and is more likely to be associated with driver events [3]. We examined recurrence from two perspectives: recurrence at the exact same site and recurrence in the same regulatory element. First, we classified COSMIC regulatory somatic variants [41] as same-site recurrent or non-recurrent (Material and methods) [25]. Our method scores recurrent variants higher than non-recurrent ones (Wilcoxon rank-sum test: *P* value <2.2 e-16; Figure 4A). Variants that occur in more than two samples have higher scores than those that are in two samples. Next we evaluated variants in recurrent regulatory elements using a separate dataset. We ran our pipeline on 119 breast cancer samples [38] and classified variants as occurring in recurrent elements or not (Material and methods). We found that variants in recurrent elements get significantly higher scores (Wilcoxon rank-sum test: *P* value <2.2e-16; Figure 4B) than variants elsewhere. Similar patterns are observed with other cancer types (Additional file 1: Figure S5). Results from CADD and GWAVA are shown in Additional file 1: Figure S6.

We note that cancer is a very heterogeneous disease and distinct molecular subtypes may involve unique oncogenic mechanisms. Thus, tumor samples from different patients may involve different driver events. These unique drivers would not show recurrence across samples. Furthermore, in the absence of large sample sizes, it might be impossible to detect recurrence of mutations. Our method would be especially useful in such scenarios, since it has the ability to prioritize deleterious variants in each tumor genome. Moreover, the functional relevance and hence the biological mechanism by which drivers act is largely unknown. Our method provides an in-depth annotation of such variants, including the relative contribution of each feature to its deleteriousness. This knowledge would greatly help understand the potential oncogenic mechanisms.

#### Germline pathogenic variants

Disease studies have identified many noncoding pathogenic variants. Designed primarily for somatic mutations, our framework contains several features that are applicable to germline variants. We tested the ability of our method to distinguish germline pathogenic variants from neutral ones. We also did possible comparisons with other germline variant prioritization tools [24,25]. We obtained pathogenic regulatory variants from HGMD [46] and three sets of controls from *Ritchie et al.* [25]: ‘unmatched’; ‘matched TSS’; and ‘matched region’ (Material and methods). Our method scored HGMD variants higher than all controls, with AUC scores of 0.86 (for ‘unmatched’), 0.74 (for ‘matched TSS’), and 0.62 (for ‘matched region’) (Figure 4C and 4D). Results from CADD [24] using the same dataset are shown in Additional file 1: Figure S7 (AUC scores: 0.75 (‘unmatched’), 0.68 (‘matched TSS’), and 0.61 (‘matched region’)). As negative sets are much larger than positive set, one concern with AUC scores is that the prediction power may come from the ability to predict negatives instead of positives. Thus we examined precision and recall to capture the ability of our method to predict positives (Additional file 1: Figure S8). Generally speaking, our method has good prediction power for pathogenic regulatory variants. In addition, GWAS SNPs show higher scores than matched common polymorphisms (mean values: 0.41 vs. 0.34, *P* value <2.2e-16; Material and methods; Additional file 1: Figure S9).

### A case study: somatic variants from an individual tumor genome

High recurrence of the *TERT* promoter mutations indicates their important roles in tumorigenesis [7]. Among the 570 cancer samples we collected, seven samples contain the *TERT* promoter mutation (chr5: 1295228). We used one Medulloblastoma sample as an example to prioritize regulatory variants from whole-genome sequencing. Among the 2,183 somatic single nucleotide variants, the *TERT* promoter mutation ranks second (0.09%). Our method further suggests potential functional impact of this variant. As shown in Table 2, this mutation occurs in ENCODE regulatory regions, creates a novel ETS binding motif and potentially affects a highly connected and known cancer gene - *TERT*. It is also found in another five liver samples and 54 COSMIC samples in our recurrence database. Besides DNA sequences, epigenome or transcriptome could also be altered in cancer genomes. These data provide sample-specific activation or inactivation signatures of regulatory sequences. If provided, our framework is flexible in integrating those data into our annotation scheme (refer to Additional file 1 for details).

**Table 2:** **Output for the** ***TERT*** **promoter mutation in a medulloblastoma sample**

**Variant**	**GERP**	**Functional annotations**	**Gain of motif**	**Associated gene**	**Network**	**Recurrence in samples**	**Recurrence database**	**Score**
chr5: 1295228	-1.46	DHS, Enhancer, TFP (E2F6, EGR1, ELF1, GABPA, HDAC2, MAX, MYC, SIN3A, TCF12, USF1, ZBTB7A, ZEB1)	Motif: Ets_known10	*TERT* (promoter) (Cancer gene)	Protein-protein interaction Centrality: 0.798	2/100 Medulloblastoma samples	5/88 Liver samples; 54 COSMIC samples	2.69
*G* → *A*			Position: 1295223 – 1295229					
			Strand: +					
			Score: 1.893 → 5.743					

We also applied CADD and GWAVA on the Medulloblastoma sample. CADD ranked the *TERT* promoter mutation 224th (10.3%) and GWAVA ranked it 25th (1.15%) with the matched region model (Additional file 1: Figure S10). However, only our method shows that the mutation corresponds to gain-of an ETS binding motif in the promoter of a cancer driver gene. Results of the additional six samples are shown in Additional file 1: Table S4.

### Output format and performance

FunSeq2 is a Linux/Unix-based tool with a web-server available at [47]. The code is also posted under GitHub [48]. It takes VCF- or BED-formatted cancer variants and generates results in either BED or VCF format (refer to Additional file 1 for examples). Users can retrieve or visualize results in concise tables through the web interface. We also provide pre-computed scores for all possible regulatory SNVs of GRCh37/hg19 on our website (the ‘core scores’ in Figure 2).

FunSeq2 runs in a tiered fashion. Building data context from bulk of data resources is time-consuming. Currently it takes about 1 week (on approximately 20 4-core 3.00-Ghz 16GB RAM PowerEdge 1955 nodes) to rebuild the data context based on pre-processed genomics data, such as ENCODE peak calls. The data context will be updated regularly to keep it up-to-date. Users can input additional data to customize the data context upon the existing one. Variant prioritization step is quite efficient. It takes about 2 to 3 min to prioritize one genome with thousands of variants on a QEMU Virtual CPU version (cpu64-rhel6) @ 2.24-GHz 1 processor Linux PC with 20 GB RAM, and a 500 GB local disk. Time comparison with CADD and GWAVA is in Additional file 1: Table S5 (our method is two times faster with equal number of variants). In addition, we implemented parallel-processing fork manager for efficient memory utilization to tackle multiple genomes in a single run. With a flexible and modularized structure, researchers can restructure the pipeline to incorporate more data and new features.

## Conclusions

We have developed a method integrating various genomic and cancer resources to prioritize cancer somatic variants, especially regulatory noncoding mutations. User data can be easily integrated into the framework. We believe that the software will be useful for researchers to identify a few somatic events among thousands for further in-depth analysis to understand the mechanisms underlying oncogenesis.

## Material and methods

### Data resources

We collected polymorphisms from 1000 Genomes Project Phase 1 [34], GERP scores and ultra-conserved elements from [31,33], sensitive/ultra-sensitive regions from [26], functional genomics data from ENCODE [15], highly occupied regions (HOT) from [27], and histone modifications ChIP-Seq and gene expression RNA-Seq data from REMC [49]. Cancer driver genes are the union of genes from *Vogelstein et al*., cancer gene consensus, and COSMIC [2,41,42]. DNA repair and actionable genes are from [43,44]. Binary protein-protein interaction network is from InWeb [50] and HINT [51]. Regulatory and phosphorylation networks are obtained from *Gerstein et al.* [35], and *Lin et al.* [37], respectively. Whole-genome somatic alterations contain 506 cancer genomes from *Alexandrov et al.* [38] and 64 prostate cancer samples from [39,40].

### High-impact variants in motifs: nucleotide resolution effect

User-input variants are first filtered against natural polymorphisms based on user-defined minor allele frequency (MAF) threshold to get rid of unlikely somatic variants (hg19). Currently, SNVs and small indels (<=20 bp) will be analyzed.

#### Motif breaking

When variants hit transcription factor binding motifs under ENCODE Chip-Seq peaks, we examined their motif breaking or conserving effect using position weight matrixes (PWM). Motif-breaking events are defined as variants decreasing the PWM scores, whereas motif-conserving events are those that do not change or increase the PWM scores [29] (we calculated the difference between mutated and germline alleles in the PWMs). Variants causing motif-breaking events are reported in the output together with the corresponding PWM changes. Transcription factor PWMs are obtained from ENCODE project [15], including TRANSFAC and JASPAR motifs.

#### Motif gaining

Whole-genome motif scanning generally discovers millions of motifs, of which a large fraction are false positives. We focused on variants occurring in promoters (defined as -2.5 kb from transcription starting sites) or regulatory elements significantly associated with genes. For each variant, +/- 29 bp are concatenated from the human reference genome (motif length is generally <30 bp). For each PWM, we scanned the 59 bp sequence. For each candidate motif encompassing the variant, we evaluated the sequence scores using TFM-Pvalue [30] (with respect to the PWM). Given a particular PWM (frequencies are transformed to log likelihoods), sequence score is computed by summing up the relevant values at each position in the PWM. If the *P* value with mutated allele < = 4e-8 and the *P* value with germline allele >4e-8, we define the variant creating a novel motif. The process is repeated for all PWMs and all variants. The sequence score changes are reported in the output.

### Associating regulatory elements with likely target genes

We define both proximal and distal associations. For proximal associations, we assign variants in gene promoters, introns, or UTRs to their nearby genes. For distal associations, in addition to those identified in [27], we further expanded the method to all ENCODE regulatory elements and identified roughly 2,448 K significant associations between 1,279 K regulatory elements and 17 K genes (see below). The distributions of regulatory element-gene associations are shown in Additional file 1: Figure S1. The median number of associations is 26 per gene and 1 per regulatory element, respectively. The association confidence is reported in the output for each regulatory element - target gene pair.

### Correlating histone modifications with gene-expression data to identify likely target genes of regulatory elements

#### Definition of distal regulatory modules (DRMs)

We started with a list of regulatory regions from three different types, namely transcription factor binding peaks (TFP), DNase I hypersensitive sites (DHS), and Segway/ChromHMM-predicted enhancers. All regulatory regions at least 1 kb from the closest gene according to the Gencode v7 annotation [52] were defined as distal regulatory modules (DRM).

#### Identifying potential regulatory targets of each DRM

We grouped different transcripts of a gene sharing the same transcription start site as a transcription start site expression unit (tssEU). For each DRM, we first considered all tssEUs within 1 Mb from it as its candidate targets. We then correlated some activity/inactivity signals at a DRM and the expression of its candidate target tssEUs, and called the ones with significant correlation values as potential DRM-target pairs as follows.

At the DRMs, we considered the enhancer marks H3K4me1 and H3K27ac as two types of activity signals, and DNA methylation as an inactivity signal. The activity level of each DRM was defined as the number of sequencing reads aligned to the DRM from the corresponding ChIP-seq experiments. The methylation level of a DRM was defined as follows. For each CpG site *i* within a DRM, we counted the number of reads that support the methylation of it (*m*_*i*_), and the total number of reads covering it (*n*_*i*_). The methylation level of the DRM was then defined as the ratio of their sums across all CpG sites in the DRM, $\frac{{\displaystyle {\sum}_i{m}_i}}{{\displaystyle {\sum}_i{n}_i}}$. For each tssEU, we defined its expression level as the number of RNA-seq reads aligned to the (TSS-50, TSS + 50) window. Both the activity signal levels and gene expression levels were normalized by the total reads, then multiplied by one million to keep them within an easily readable range of values.

We collected all bisulfite sequencing, ChIP-Seq, and RNA-Seq data from the Roadmap Epigenomics project website [49] (EDACC release 9^1^). We considered 19 tissue types with data for both the activity signals and gene expression, and 20 tissue types with data for both the inactivity signal and gene expression. For RNA-seq, we used the paired-end 100 bp Poly-A enriched datasets. For experiments with replicates, we used the mean value across the replicates as the expression level of a gene.

For each DRM-candidate target pair, we computed the correlations of their activity/inactivity and expression levels across the different tissue types. We computed both value-based Pearson correlation and rank-based Spearman correlation. The statistical significance of each correlation value was evaluated by computing a *P* value based on one-tailed tests using the built-in functions in R. Briefly, for Pearson correlation, the correlation values would follow a *t* distribution with *n* - 2 degrees of freedom (where *n* is the number of tissue types) if the samples are drawn independently from normal distributions. The Fisher’s Z transformation was used to compute the *P* values. For Spearman correlation, the *P* value was computed based on a procedure proposed by Hollander and Wolfe [53]. For activity signals, we considered the right tail, which means we looked for correlations significantly more positive than would be expected by chance. For inactivity signals, we considered the left tail, which means we looked for correlations significantly more negative (that is, strong anti-correlations) than would be expected by chance. All *P* values were then adjusted for multiple hypotheses testing using the Bonferroni, Holm, Benjamini-Hochberg (BH), or Benjamini-Yekutieli (BY) methods.

### Differential gene expression analysis

We incorporated a module to detect differentially expressed genes in cancer samples (relative to matched normal) from RNA-Seq data. When provided with gene expression files, our module calls NOISeq [54] when having RPKM values and DESeq [55] with raw read counts (from reads-mapping tools) to detect differentially expressed genes. Genes that are up- or down-regulated with FDR <0.05 (with biological replicates) and FDR <0.1 (without replicates) in cancer samples are identified and annotated in the output.

### Network analysis of variants associated with genes

For each variant associated with genes, we examined its topological properties in various networks. For each network, we calculated the centrality position (cumulative probability after ordering centralities of all genes increasingly) of the associated gene. If one variant is associated with multiple genes or the associated gene participates in multiple networks, the maximum cumulative probability is used as the continuous value for centrality score. Scripts are provided to calculate network centralities (Additional file 1). Users can easily incorporate other networks in this analysis.

### Recurrence database from whole-genome sequencing

With the increasing number of cancer samples being whole-genome sequenced, we are able to study recurrence patterns in regulatory sequences. We analyzed somatic alterations from 570 samples of 10 cancer types to create the recurrence database [38–40], similar to the cancer recurrent gene database in cBio [56]. For each cancer type, recurrent regulatory elements, coding genes, and the same-site mutations are stored as entries in the database. We also incorporated same-site recurrent regulatory variants from COSMIC (version 68) into our database. Recurrent elements are defined as identified in whole-genome sequencing and observed in at least two samples.

### Weighted scoring scheme

#### Coding scoring scheme

Variants in coding regions (GENCODE 16 for the current version; users can replace this with other GENECODE versions) are analyzed with VAT (variant annotation tool) [57]. Variants are ranked based on the following scheme (each criterion gets score 1): (1) non-synonymous; (2) premature stop; (3) is the gene under strong selection; (4) is the gene a network hub; (5) recurrent; (6) GERP score >2.

#### Noncoding scoring scheme (weighted scoring scheme)

Features used to score noncoding variants are shown in Additional file 1: Table S3. In general, features can be classified into two classes: discrete and continuous. Discrete features are binary, such as in ultra-conserved elements or not. Continuous features: (1) GERP score; (2) motif-breaking score is the difference between germline and mutated alleles in PWMs; (3) motif-gaining score is the sequence score difference between mutated and germline alleles; (4) network centrality score (the cumulative probability, see ‘Network analysis of variants associated with genes’). If one variant has multiple values of a particular feature (for example, breaking multiple motifs), the largest value is used.

We weighted each feature based on the mutation patterns observed in the 1000 Genomes polymorphisms. We randomly selected 10% of the 1000 Genomes Phase 1 SNPs (approximately 3.7 M) and ran them through our pipeline. For each discrete feature *d*, we calculated the probability *p*_*d*_ that overlaps a natural polymorphism. Then we computed 1-Shannon entropy (1) as its weighted value *w*_*d*_. The value ranges from 0 to 1 and is monotonically decreasing when the probability is between 0 and 0.5c below 0.5).

1 $$ {w}_d=1+{p}_dlo{g}_2{p}_d+\left(1-{p}_d\right)lo{g}_2\left(1-{p}_d\right) $$

$$ {p}_d=\frac{number\  of\  polymorphisms\  with\  feature\ d}{total\  number\  of\  polymorphisms} $$

The situation is more complex for continuous features, as different feature values have different probabilities of being observed in natural polymorphisms. Thus, one weight cannot suffice for varied feature values. For a continuous feature *c*, which is associated with a score *v*_*c*_ (for example, motif-breaking score), we calculated feature weights for each *v*_*c*_. In particular, we discretized at each *v*_*c*_ and computed 1-Shannon entropy using (2). Then we fitted a smooth curve for all *v*_*c*_ to obtain continuous ${w}_c^{v_c}$. Now, when we come to evaluate the continuous feature *c* for a particular variant, we calculate its weighted value (on the curve) using the actual *v*_*c*_ corresponding to the variant.

2 $$ {w}_c^{v_c}=1+{p}_c^{\ge {v}_c}lo{g}_2{p}_c^{\ge {v}_c}+\left(1-{p}_c^{\ge {v}_c}\right)lo{g}_2\left(1-{p}_c^{\ge {v}_c}\right) $$

$$ {p}_c^{\ge {v}_c}=\frac{number\  of\  polymorphisms\  with\  score\ge {v}_c\ for\  feature\ c}{total\  number\  of\  polymorphisms} $$

Taking ‘motif-breaking score’ as an example (Figure 3B), for each score *v*, we calculated the probability of observing motif-breaking scores ≥ *v* in polymorphism data, then used (2) to fit the smooth function. ‘nls’ function in R is used to fit curves.

The criterion of ‘GERP >2’ has been commonly used to define conserved bases [15]. For the GERP score, we chose to use a sigmoid transformation to transform scores to the range 0 to 1. The parameters we chose make the sigmoid curve sharp at ‘GERP = 2’ (Additional file 1: Figure S2). The sigmoid transformation preserves the ‘GERP > 2’ cutoff and makes the score continuous at the same time. We calculated (1) treating ‘GERP >2’ as a discrete feature. Then we used *w*_*d*_ * *sigmoid transformed value* to assign weighted value for each continuous GERP score.

Finally, for each cancer variant, we scored it by summing the weighted values of all its features (3). If a particular feature is not observed, it is not used in the scoring. Considering the situation that some features are subsets of other features, to avoid overweighting similar features, we took into account feature dependencies when calculating the summed scores. As shown in Additional file 1: Table S3, when having leaf features, the weighted values of root features are ignored. For example, when a variant occurs in sensitive regions, the score of ‘in functional annotations’ is not used in the sum-up. Leaf features are assumed independent. Variants ranked on top of the output are those with higher scores and are most likely to be deleterious.

3 $$ score={\displaystyle \sum_d{w}_d}+{\displaystyle \sum_c{w}_c^{v_c}} $$

### Application to regulatory pathogenic and cancer somatic variants

All scores in this section are ‘core scores’ described in Figure 2.

#### Same-site recurrent somatic variants

We obtained noncoding somatic variants from COSMIC (version 68). Recurrent variants (10,041) are defined as identified in whole-genome sequencing and observed in at least two samples. All other variants (1,311,389) are non-recurrent ones. After excluding variants in coding regions (GENCODE 16) and mitochondrion, there are 956 variants occurring in more than two samples, 8,932 variants in two samples, and 1,305,699 non-recurrent variants. Because the same sample from different papers may have multiple ids, we also defined recurrence based on number of studies. Study-based recurrent variants also have higher scores than non-recurrent ones (Wilcoxon test: *P* value = 7.8e-08).

As we know, COSMIC collects somatic alterations from diverse papers and studies. We noticed a potential artifact related to pseudogenes (Additional file 1: Figure S3) - the percentage of variants in pseudogenes increases with the number of recurrent samples or studies. After removing these variants, the trend of prediction scores persists (Additional file 1: Figure S4).

#### Somatic variants in recurrent regulatory elements

Regulatory regions mutated in more than one sample are defined as recurrent regulatory elements, such as the same TF binding motif or the same noncoding RNA. We first identified recurrent regulatory elements across multiple cancer samples. Then we classified variants either in recurrent regulatory elements or not. As recurrent regulatory elements are functional annotations, to make a fair comparison, we filtered variants in non-recurrent regulatory elements as those also in functional annotations. From 119 breast cancer samples, there are 24,022 (4,841 in recurrent elements mutated in more than two samples; 19,181 in elements mutated in two samples) and 126,217 variants in recurrent and non-recurrent regulatory elements, respectively. The feature of recurrence is not considered in the weighted scoring scheme for variants in sections 1 and 2.

#### Germline pathogenic variants and matched controls

Genome locations of pathogenic regulatory variants (from HGMD [46] -1,614) and three sets of negative controls were downloaded from GWAVA [25]. ‘Unmatched’ control consists of 161,400 likely neutral polymorphisms randomly selected from 1000 Genomes Phase1 with allele frequency > =1%. Restrictions of ‘2Kb around TSS’ and ‘1Kb around HGMD variants’ are applied to ‘matched TSS’ and ‘matched region’ controls, respectively. ‘Matched TSS’ control includes 16,140 variants and ‘matched region’ control has 5,027 variants. Allele information for HGMD variants was obtained from HGMD database (1,527 variants). For controls, the alleles were from ENSEMBL BioMart, using reference SNP ids. We then excluded polymorphisms that were in coding regions or used in the weighted scoring scheme, from controls (‘matched region’ - 4,258; ‘matched TSS’ - 13,861; ‘unmatched’ - 144,086).

We downloaded pre-calculated CADD scores for 1000 Genomes variants and extracted corresponding scores for control sets. For HGMD variants, we used the online CADD server to obtain the scores.

We compared the prediction scores of HGMD variants with three sets of controls using various measures - TPR (true positive rate), FPR (false positive rate), precision, and recall. We treated HGMD as positive set and controls as negative sets. For each possible score, we draw the cutoff to make predictions and calculated - TPR = TP/(TP + FN); FPR = FP/(FP + TN); Precision = TP/(TP + FP); Recall = TP/(TP + FN) (TP: true positive; FP: false positive; TN: true negative; FN: false negative). AUC score is the cumulative area under the curve of TPR and FPR.

We also tested our method with GWAS SNPs (6,604) and matched controls (66,040) from [25]. Allele information was obtained from ENSEMBL BioMart.

### Framework flexibility

User data can be easily incorporated into our framework. Cancer sample-specific studies, such as histone modifications and gene expression, are especially useful in evaluating variants’ impact. Please refer to ‘Additional file 1’ for usage.

## Additional file

Additional file 1: Figure S1: Distribution of linkages between regulatory elements and genes. **Figure S2:** Weighted values for continuous features. **Figure S3:** Percentage of variants in pseudogene increases as the number of recurrent samples/studies increases. We suspected that reads containing these variants should probably be mapped to parent genes of pseudogene, instead of the noncoding genome. **Figure S4:** After excluding variants in pseudogenes, the trend of prediction scores persists. **Figure S5:** Prediction scores of variants in recurrent regulatory elements from 88 liver cancer samples. **Figure S6:** Comparisons with GWAVA and CADD using breast cancer variants. **Figure S7:** ROC curves comparing HGMD with controls using CADD. **Figure S8:** Precision and recall comparing HGMD with controls. **Figure S9:** Prediction scores of GWAS SNPs and matched control. **Figure S10:** Relationship between distance to TSS and prediction scores (using variants from one Medulloblastoma sample - MB59). Red dot is the *TERT* promoter mutation. We reported ‘matched region’ model of GWAVA for all analysis, as the model is less prone to bias. **Table S1:** Gain-of-motif of the *TERT* promoter mutations (motif name # motif start coordinate # motif end coordinate # motif strand # variant position # alternative sequence score # germline sequence score). **Table S2:** Features used to annotate variants. **Table S3:** Weighted scoring scheme. **Table S4:** Rankings of the *TERT* promoter mutation in seven cancer samples. ‘Matched region’ model is used for GWAVA (Figure S10). **Table S5:** Time comparisons using about 2,000 variants.

## Abbreviations

COSMIC: Catalogue of Somatic Mutations in Cancer
ENCODE: The Encyclopedia of DNA Elements
GWAS: Genome-wide association studies
HGMD: The Human Gene Mutation Database
PWM: Position weight matrix
REMC: Roadmap Epigenomics Mapping Consortium
SNP: Single nucleotide polymorphisms
TERT: Telomerase reverse transcriptase
TF: Transcription factor
TSS: Transcription starting site

## References

[1] Greenman C, Stephens P, Smith R, Dalgliesh GL, Hunter C, Bignell G, Davies H, Teague J, Butler A, Stevens C, Edkins S, O'Meara S, Vastrik I, Schmidt EE, Avis T, Barthorpe S, Bhamra G, Buck G, Choudhury B, Clements J, Cole J, Dicks E, Forbes S, Gray K, Halliday K, Harrison R, Hills K, Hinton J, Jenkinson A, Jones D (2007). Patterns of somatic mutation in human cancer genomes. Nature.

[2] Futreal PA, Coin L, Marshall M, Down T, Hubbard T, Wooster R, Rahman N, Stratton MR (2004). A census of human cancer genes. Nat Rev Cancer.

[3] Dees ND, Zhang Q, Kandoth C, Wendl MC, Schierding W, Koboldt DC, Mooney TB, Callaway MB, Dooling D, Mardis ER, Wilson RK, Ding L (2012). MuSiC: identifying mutational significance in cancer genomes. Genome Res.

[4] Reimand J, Bader GD (2013). Systematic analysis of somatic mutations in phosphorylation signaling predicts novel cancer drivers. Mol Syst Biol.

[5] Tamborero D, Gonzalez-Perez A, Lopez-Bigas N (2013). OncodriveCLUST: exploiting the positional clustering of somatic mutations to identify cancer genes. Bioinformatics.

[6] Tamborero D, Gonzalez-Perez A, Perez-Llamas C, Deu-Pons J, Kandoth C, Reimand J, Lawrence MS, Getz G, Bader GD, Ding L, Lopez-Bigas N (2013). Comprehensive identification of mutational cancer driver genes across 12 tumor types. Sci Rep.

[7] Huang FW, Hodis E, Xu MJ, Kryukov GV, Chin L, Garraway LA (2013). Highly recurrent TERT promoter mutations in human melanoma. Science.

[8] Horn S, Figl A, Rachakonda PS, Fischer C, Sucker A, Gast A, Kadel S, Moll I, Nagore E, Hemminki K, Schadendorf D, Kumar R (2013). TERT promoter mutations in familial and sporadic melanoma. Science.

[9] Killela PJ, Reitman ZJ, Jiao Y, Bettegowda C, Agrawal N, Diaz LA, Friedman AH, Friedman H, Gallia GL, Giovanella BC, Grollman AP, He TC, He Y, Hruban RH, Jallo GI, Mandahl N, Meeker AK, Mertens F, Netto GJ, Rasheed BA, Riggins GJ, Rosenquist TA, Schiffman M, Shih Ie M, Theodorescu D, Torbenson MS, Velculescu VE, Wang TL, Wentzensen N, Wood LD (2013). TERT promoter mutations occur frequently in gliomas and a subset of tumors derived from cells with low rates of self-renewal. Proc Natl Acad Sci U S A.

[10] Vinagre J, Almeida A, Populo H, Batista R, Lyra J, Pinto V, Coelho R, Celestino R, Prazeres H, Lima L, Melo M, da Rocha AG, Preto A, Castro P, Castro L, Pardal F, Lopes JM, Santos LL, Reis RM, Cameselle-Teijeiro J, Sobrinho-Simoes M, Lima J, Maximo V, Soares P (2013). Frequency of TERT promoter mutations in human cancers. Nat Commun.

[11] Maurano MT, Humbert R, Rynes E, Thurman RE, Haugen E, Wang H, Reynolds AP, Sandstrom R, Qu H, Brody J, Shafer A, Neri F, Lee K, Kutyavin T, Stehling-Sun S, Johnson AK, Canfield TK, Giste E, Diegel M, Bates D, Hansen RS, Neph S, Sabo PJ, Heimfeld S, Raubitschek A, Ziegler S, Cotsapas C, Sotoodehnia N, Glass I, Sunyaev SR (2012). Systematic localization of common disease-associated variation in regulatory DNA. Science.

[12] Grossman SR, Andersen KG, Shlyakhter I, Tabrizi S, Winnicki S, Yen A, Park DJ, Griesemer D, Karlsson EK, Wong SH, Cabili M, Adegbola RA, Bamezai RN, Hill AV, Vannberg FO, Rinn JL, Genomes P, Lander ES, Schaffner SF, Sabeti PC (2013). Identifying recent adaptations in large-scale genomic data. Cell.

[13] Sakabe NJ, Savic D, Nobrega MA (2012). Transcriptional enhancers in development and disease. Genome Biol.

[14] Ward LD, Kellis M (2012). Interpreting noncoding genetic variation in complex traits and human disease. Nat Biotechnol.

[15] The Encode Project Consortium (2012). An integrated encyclopedia of DNA elements in the human genome. Nature.

[16] Lowe CB, Haussler D (2012). 29 mammalian genomes reveal novel exaptations of mobile elements for likely regulatory functions in the human genome. PLoS One.

[17] Schaub MA, Boyle AP, Kundaje A, Batzoglou S, Snyder M (2012). Linking disease associations with regulatory information in the human genome. Genome Res.

[18] Ward LD, Kellis M (2012). HaploReg: a resource for exploring chromatin states, conservation, and regulatory motif alterations within sets of genetically linked variants. Nucleic Acids Res.

[19] Boyle AP, Hong EL, Hariharan M, Cheng Y, Schaub MA, Kasowski M, Karczewski KJ, Park J, Hitz BC, Weng S, Cherry JM, Snyder M (2012). Annotation of functional variation in personal genomes using RegulomeDB. Genome Res.

[20] Wang K, Li M, Hakonarson H (2010). ANNOVAR: functional annotation of genetic variants from high-throughput sequencing data. Nucleic Acids Res.

[21] Paila U, Chapman BA, Kirchner R, Quinlan AR (2013). GEMINI: integrative exploration of genetic variation and genome annotations. PLoS Comput Biol.

[22] Coetzee SG, Rhie SK, Berman BP, Coetzee GA, Noushmehr H (2012). FunciSNP: an R/bioconductor tool integrating functional non-coding data sets with genetic association studies to identify candidate regulatory SNPs. Nucleic Acids Res.

[23] McLaren W, Pritchard B, Rios D, Chen Y, Flicek P, Cunningham F (2010). Deriving the consequences of genomic variants with the Ensembl API and SNP Effect Predictor. Bioinformatics.

[24] Kircher M, Witten DM, Jain P, O'Roak BJ, Cooper GM, Shendure J (2014). A general framework for estimating the relative pathogenicity of human genetic variants. Nat Genet.

[25] Ritchie GR, Dunham I, Zeggini E, Flicek P (2014). Functional annotation of noncoding sequence variants. Nat Methods.

[26] Khurana E, Fu Y, Colonna V, Mu XJ, Kang HM, Lappalainen T, Sboner A, Lochovsky L, Chen J, Harmanci A, Das J, Abyzov A, Balasubramanian S, Beal K, Chakravarty D, Challis D, Chen Y, Clarke D, Clarke L, Cunningham F, Evani US, Flicek P, Fragoza R, Garrison E, Gibbs R, Gumus ZH, Herrero J, Kitabayashi N, Kong Y, Lage K (2013). Integrative annotation of variants from 1092 humans: application to Cancer Genomics. Science.

[27] Yip KY, Cheng C, Bhardwaj N, Brown JB, Leng J, Kundaje A, Rozowsky J, Birney E, Bickel P, Snyder M, Gerstein M (2012). Classification of human genomic regions based on experimentally determined binding sites of more than 100 transcription-related factors. Genome Biol.

[28] Kheradpour P, Ernst J, Melnikov A, Rogov P, Wang L, Zhang X, Alston J, Mikkelsen TS, Kellis M (2013). Systematic dissection of regulatory motifs in 2000 predicted human enhancers using a massively parallel reporter assay. Genome Res.

[29] Mu XJ, Lu ZJ, Kong Y, Lam HY, Gerstein MB (2011). Analysis of genomic variation in non-coding elements using population-scale sequencing data from the 1000 Genomes Project. Nucleic Acids Res.

[30] Touzet H, Varre JS (2007). Efficient and accurate P-value computation for Position Weight Matrices. Algorithms Mol Biol.

[31] Bejerano G, Pheasant M, Makunin I, Stephen S, Kent WJ, Mattick JS, Haussler D (2004). Ultraconserved elements in the human genome. Science.

[32] Thomas MA, Weston B, Joseph M, Wu W, Nekrutenko A, Tonellato PJ (2003). Evolutionary dynamics of oncogenes and tumor suppressor genes: higher intensities of purifying selection than other genes. Mol Biol Evol.

[33] Cooper GM, Stone EA, Asimenos G, Program NCS, Green ED, Batzoglou S, Sidow A (2005). Distribution and intensity of constraint in mammalian genomic sequence. Genome Res.

[34] The 1000 Genomes Project Consortium (2012). An integrated map of genetic variation from 1,092 human genomes. Nature.

[35] Gerstein MB, Kundaje A, Hariharan M, Landt SG, Yan KK, Cheng C, Mu XJ, Khurana E, Rozowsky J, Alexander R, Min R, Alves P, Abyzov A, Addleman N, Bhardwaj N, Boyle AP, Cayting P, Charos A, Chen DZ, Cheng Y, Clarke D, Eastman C, Euskirchen G, Frietze S, Fu Y, Gertz J, Grubert F, Harmanci A, Jain P, Kasowski M (2012). Architecture of the human regulatory network derived from ENCODE data. Nature.

[36] Goh KI, Cusick ME, Valle D, Childs B, Vidal M, Barabasi AL (2007). The human disease network. Proc Natl Acad Sci U S A.

[37] Lin J, Xie Z, Zhu H, Qian J (2010). Understanding protein phosphorylation on a systems level. Brief Funct Genomics.

[38] Alexandrov LB, Nik-Zainal S, Wedge DC, Aparicio SA, Behjati S, Biankin AV, Bignell GR, Bolli N, Borg A, Borresen-Dale AL, Boyault S, Burkhardt B, Butler AP, Caldas C, Davies HR, Desmedt C, Eils R, Eyfjord JE, Foekens JA, Greaves M, Hosoda F, Hutter B, Ilicic T, Imbeaud S, Imielinsk M, Jager N, Jones DT, Jones D, Knappskog S, Kool M (2013). Signatures of mutational processes in human cancer. Nature.

[39] Baca SC, Prandi D, Lawrence MS, Mosquera JM, Romanel A, Drier Y, Park K, Kitabayashi N, MacDonald TY, Ghandi M, Van Allen E, Kryukov GV, Sboner A, Theurillat JP, Soong TD, Nickerson E, Auclair D, Tewari A, Beltran H, Onofrio RC, Boysen G, Guiducci C, Barbieri CE, Cibulskis K, Sivachenko A, Carter SL, Saksena G, Voet D, Ramos AH, Winckler W (2013). Punctuated evolution of prostate cancer genomes. Cell.

[40] Berger MF, Lawrence MS, Demichelis F, Drier Y, Cibulskis K, Sivachenko AY, Sboner A, Esgueva R, Pflueger D, Sougnez C, Onofrio R, Carter SL, Park K, Habegger L, Ambrogio L, Fennell T, Parkin M, Saksena G, Voet D, Ramos AH, Pugh TJ, Wilkinson J, Fisher S, Winckler W, Mahan S, Ardlie K, Baldwin J, Simons JW, Kitabayashi N, MacDonald TY (2011). The genomic complexity of primary human prostate cancer. Nature.

[41] Forbes SA, Bindal N, Bamford S, Cole C, Kok CY, Beare D, Jia M, Shepherd R, Leung K, Menzies A, Teague JW, Campbell PJ, Stratton MR, Futreal PA (2011). COSMIC: mining complete cancer genomes in the Catalogue of Somatic Mutations in Cancer. Nucleic Acids Res.

[42] Vogelstein B, Papadopoulos N, Velculescu VE, Zhou S, Diaz LA, Kinzler KW (2013). Cancer genome landscapes. Science.

[43] Ruark E, Snape K, Humburg P, Loveday C, Bajrami I, Brough R, Rodrigues DN, Renwick A, Seal S, Ramsay E, Duarte Sdel V, Rivas MA, Warren-Perry M, Zachariou A, Campion-Flora A, Hanks S, Murray A, Ansari Pour N, Douglas J, Gregory L, Rimmer A, Walker NM, Yang TP, Adlard JW, Barwell J, Berg J, Brady AF, Brewer C, Brice G, Chapman C (2013). Mosaic PPM1D mutations are associated with predisposition to breast and ovarian cancer. Nature.

[44] Wagle N, Berger MF, Davis MJ, Blumenstiel B, Defelice M, Pochanard P, Ducar M, Van Hummelen P, Macconaill LE, Hahn WC, Meyerson M, Gabriel SB, Garraway LA (2012). High-throughput detection of actionable genomic alterations in clinical tumor samples by targeted, massively parallel sequencing. Cancer Discov.

[45] Heintzman ND, Hon GC, Hawkins RD, Kheradpour P, Stark A, Harp LF, Ye Z, Lee LK, Stuart RK, Ching CW, Ching KA, Antosiewicz-Bourget JE, Liu H, Zhang X, Green RD, Lobanenkov VV, Stewart R, Thomson JA, Crawford GE, Kellis M, Ren B (2009). Histone modifications at human enhancers reflect global cell-type-specific gene expression. Nature.

[46] Stenson PD, Ball EV, Mort M, Phillips AD, Shiel JA, Thomas NS, Abeysinghe S, Krawczak M, Cooper DN (2003). Human Gene Mutation Database (HGMD): 2003 update. Hum Mutat.

[47] **Web-server URL.** [http://funseq2.gersteinlab.org]

[48] **GitHub page.** [http://github.gersteinlab.org/FunSeq2]

[49] Bernstein BE, Stamatoyannopoulos JA, Costello JF, Ren B, Milosavljevic A, Meissner A, Kellis M, Marra MA, Beaudet AL, Ecker JR, Farnham PJ, Hirst M, Lander ES, Mikkelsen TS, Thomson JA (2010). The NIH roadmap epigenomics mapping consortium. Nat Biotechnol.

[50] Lage K, Karlberg EO, Storling ZM, Olason PI, Pedersen AG, Rigina O, Hinsby AM, Tumer Z, Pociot F, Tommerup N, Moreau Y, Brunak S (2007). A human phenome-interactome network of protein complexes implicated in genetic disorders. Nat Biotechnol.

[51] Das J, Yu H (2012). HINT: High-quality protein interactomes and their applications in understanding human disease. BMC Syst Biol.

[52] Harrow J, Frankish A, Gonzalez JM, Tapanari E, Diekhans M, Kokocinski F, Aken BL, Barrell D, Zadissa A, Searle S, Barnes I, Bignell A, Boychenko V, Hunt T, Kay M, Mukherjee G, Rajan J, Despacio-Reyes G, Saunders G, Steward C, Harte R, Lin M, Howald C, Tanzer A, Derrien T, Chrast J, Walters N, Balasubramanian S, Pei B, Tress M (2012). GENCODE: the reference human genome annotation for The ENCODE Project. Genome Res.

[53] Hollander M, Wolfe DA (1973). Nonparametric Statistical Methods.

[54] Tarazona S, Garcia-Alcalde F, Dopazo J, Ferrer A, Conesa A (2011). Differential expression in RNA-seq: a matter of depth. Genome Res.

[55] Anders S, Huber W (2010). Differential expression analysis for sequence count data. Genome Biol.

[56] Cerami E, Gao J, Dogrusoz U, Gross BE, Sumer SO, Aksoy BA, Jacobsen A, Byrne CJ, Heuer ML, Larsson E, Antipin Y, Reva B, Goldberg AP, Sander C, Schultz N (2012). The cBio cancer genomics portal: an open platform for exploring multidimensional cancer genomics data. Cancer Discov.

[57] Habegger L, Balasubramanian S, Chen DZ, Khurana E, Sboner A, Harmanci A, Rozowsky J, Clarke D, Snyder M, Gerstein M (2012). VAT: a computational framework to functionally annotate variants in personal genomes within a cloud-computing environment. Bioinformatics.

