# Supplementary material for: FunSeq2: a framework for prioritizing noncoding regulatory variants in cancer
Source: Genome Biol. 2014 Oct 2;15(10):480. doi: 10.1186/s13059-014-0480-5 (PMC4203974; doi:10.1186/s13059-014-0480-5)
Supplement: Additional file 1: Figure S1: — Distribution of linkages between regulatory elements and genes. Figure S2: Weighted values for continuous features. Figure S3: Percentage of variants in pseudogene increases as the number of recurrent samples/studies increases. We suspected that reads containing these variants should probably be mapped to parent genes of pseudogene, instead of the noncoding genome. Figure S4: After excluding variants in pseudogenes, the trend of prediction scores persists. Figure S5: Prediction scores of variants in recurrent regulatory elements from 88 liver cancer samples. Figure S6: Comparisons with GWAVA and CADD using breast cancer variants. Figure S7: ROC curves comparing HGMD with controls using CADD. Figure S8: Precision and recall comparing HGMD with controls. Figure S9: Prediction scores of GWAS SNPs and matched control. Figure S10: Relationship between distance to TSS and prediction scores (using variants from one Medulloblastoma sample - MB59). Red dot is the TERT promoter mutation. We reported ‘matched region’ model of GWAVA for all analysis, as the model is less prone to bias. Table S1: Gain-of-motif of the TERT promoter mutations (motif name # motif start coordinate # motif end coordinate # motif strand # variant position # alternative sequence score # germline sequence score). Table S2: Features used to annotate variants. Table S3: Weighted scoring scheme. Table S4: Rankings of the TERT promoter mutation in seven cancer samples. ‘Matched region’ model is used for GWAVA (Figure S10). Table S5: Time comparisons using about 2,000 variants. [file 13059_2014_480_MOESM1_ESM.docx]

Additional file

**FunSeq2: A framework for prioritizing noncoding regulatory variants in cancer**

Yao Fu, Zhu Liu, Shaoke Lou, Jason Bedford, Xinmeng Jasmine Mu, Kevin Y. Yip, Ekta Khurana, Mark Gerstein

**Figure S1. Distribution of linkages between regulatory elements and genes.**

**Figure S2. Weighted values for continuous features.**

#

**Figure S3.** **Percentage of variants in pseudogene increases as the number of recurrent samples/studies increases.** We suspected that reads containing these variants should probably be mapped to parent genes of pseudogene, instead of the non-coding genome.

**Figure S4.** **After excluding variants in pseudogenes, the trend of prediction scores persists.**

#

**Figure S5.** **Prediction scores of variants in recurrent regulatory elements from 88 liver cancer samples.**

**Figure S6.** **Comparisons with GWAVA and CADD using breast cancer variants.**

#
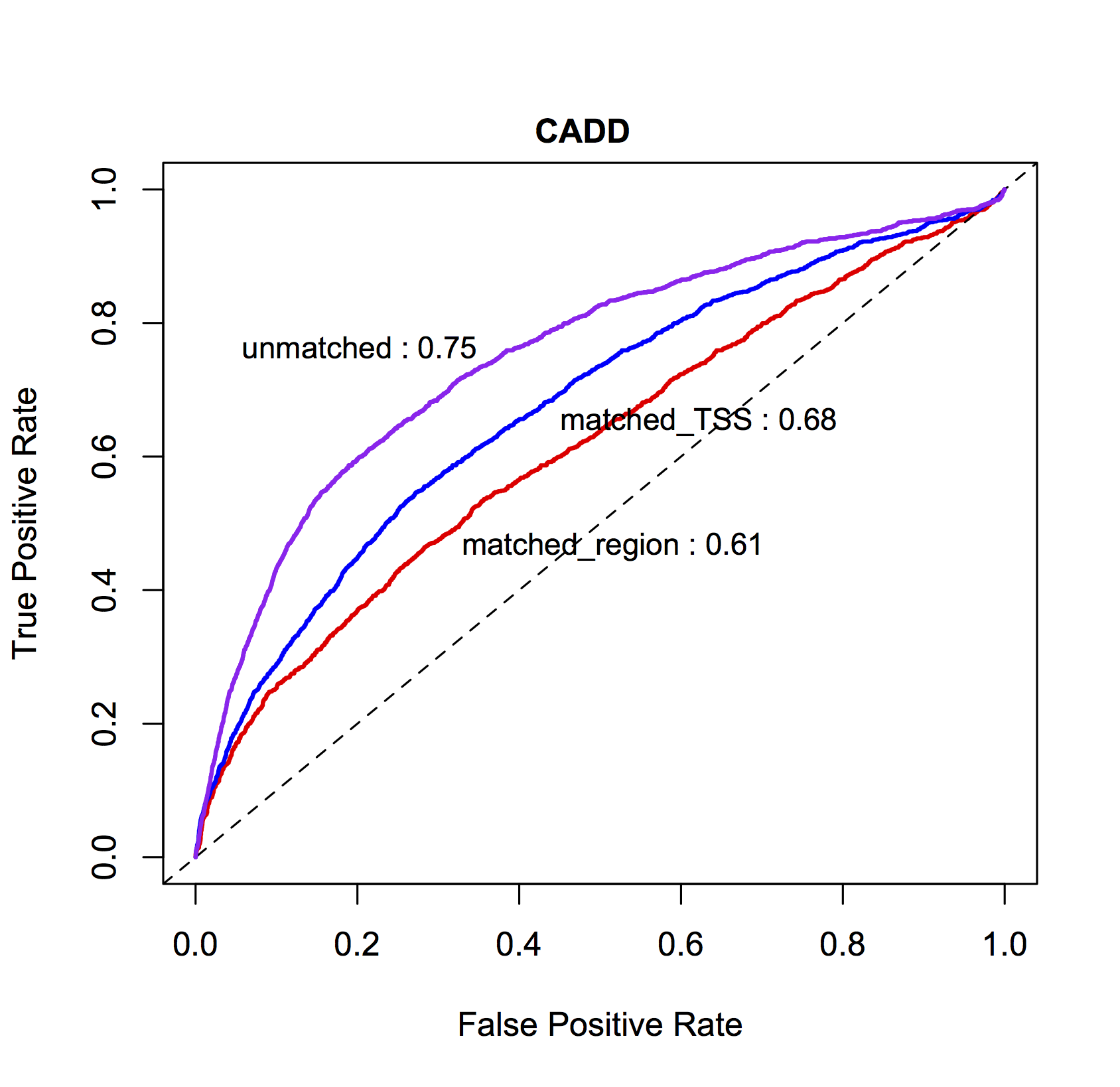


**Figure S7. ROC curves comparing HGMD with controls using CADD.**

**Figure S8. Precision and recall comparing HGMD with controls.**


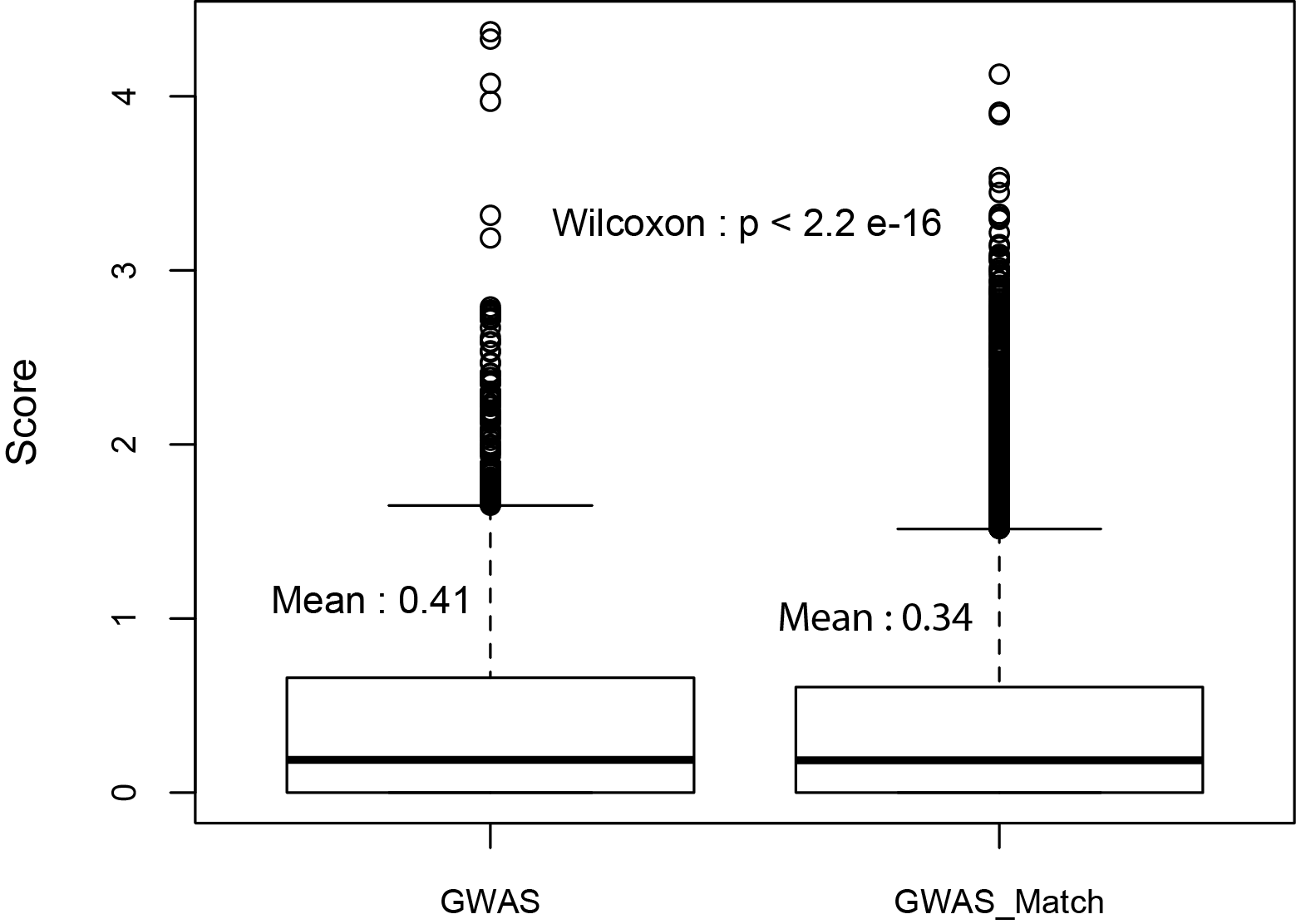


**Figure S9. Prediction scores of GWAS SNPs and matched control.**


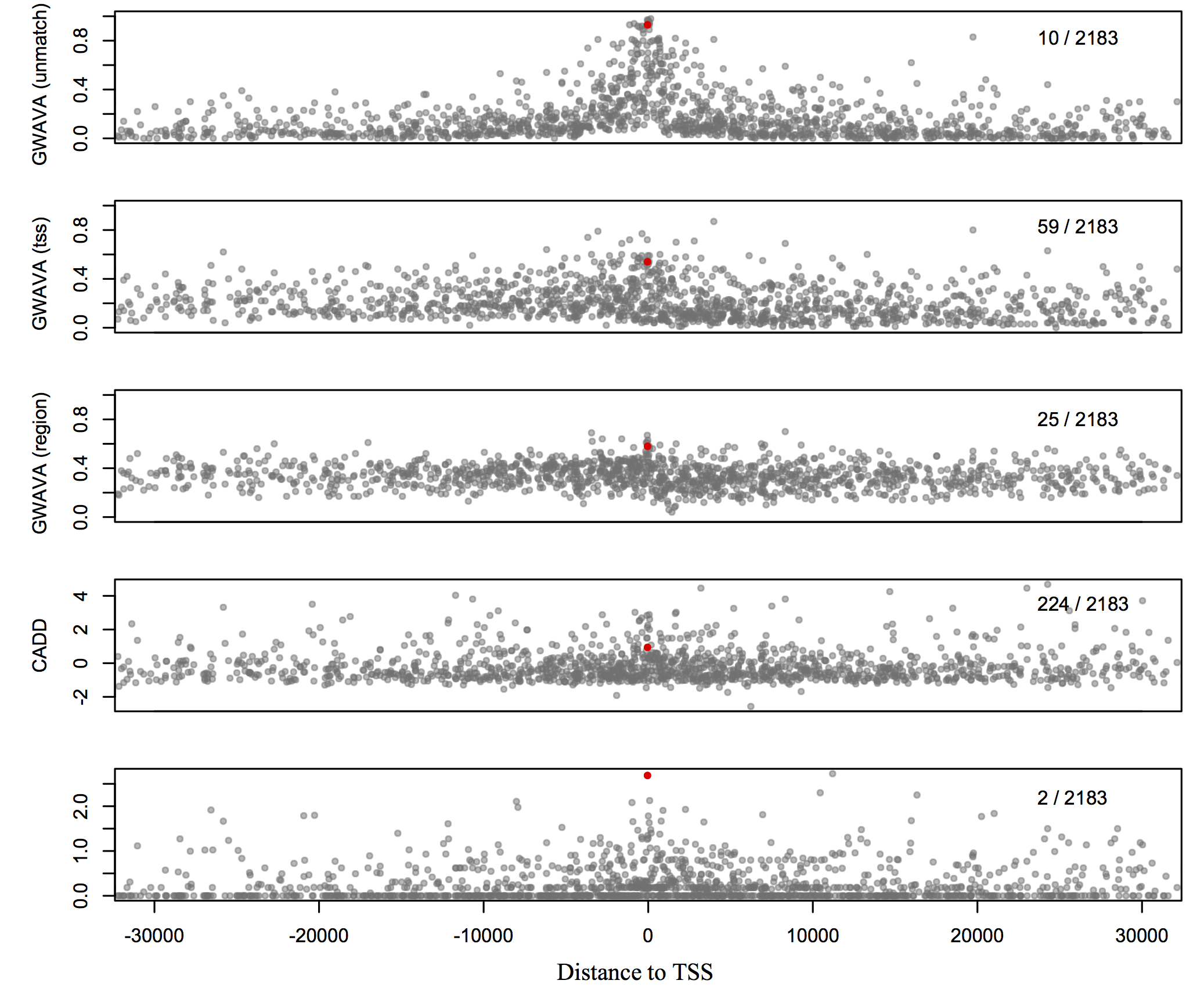


**Figure S10. Relationship between distance to TSS and prediction scores (using variants from one Medulloblastoma sample - MB59).** Red dot is the *TERT* promoter mutation. We reported ‘matched region’ model of GWAVA for all analysis, as the model is less prone to bias.

| **Mutation position** | **Gain of motif** |
| --- | --- |
| chr5 1295250 | Ets_known10#1295246#1295252#+#4#5.743#2.472 |
| chr5 1295228 | Ets_known10#1295223#1295229#+#5#5.743#1.893 |

**Table S1.** Gain-of-motif of the *TERT* promoter mutations (motif name # motif start coordinate # motif end coordinate # motif strand # variant position # alternative sequence score # germline sequence score).

| Feature | Details |
| --- | --- |
| Functional annotations | ncRNA, Pseudogenes, transcription factor binding peaks and motifs, DNaseI hypersensitive sites, enhancers (segway/chromHMM), distal regulatory modules … |
| In sensitive/ultra-sensitive regions | Human population-level conservation |
| Motif-breaking and motif-gaining | Motif position, strand, name, PWM changes |
| GERP score | GERP score |
| In ultra-conserved regions | Evolutionary conserved regions |
| In HOT regions | Transcription factor highly occupied regions, shown with corresponding cell-line information |
| Associated with genes | Variants in CDS, Intron, UTR or other regulatory elements associated with genes. The correlations and significance between histone modifications and gene expression levels are reported for regulatory elements |
| Network centrality | For variants associated with genes, we reported their network centralities in protein-protein interaction, phosphorylation, and regulatory networks |
| Gene information | Whether a gene is a cancer gene, DNA-repair gene, drug target, or differentially expressed in tumor samples … |
| Recurrence among input samples | We do recurrence analysis within input samples; recurrent variants, genes, or regulatory elements are reported together with sample info |
| Recurrence database | Variants in input samples are compared to the recurrence database. Recurrence information in 570 samples and COSMIC is reported |
| User annotations | Annotations provided by users, such as epigenetics/open chromatin profiles |

**Table S2.** Features used to annotate variants.

| Feature | Class | Weight |
| --- | --- | --- |
| In functional annotations | Discrete | 0.18521432 |
| In sensitive regions | Discrete | 0.96910593 |
| In ultra-sensitive regions | Discrete | 0.99723589 |
| Motif-breaking score | Continuous | $0.97314 +0.01863 * x$ |
| Motif-gaining score | Continuous | $0.960322+0.005138 * x$ |
| Network centrality score | Continuous | $e^{-3.231 + 3.233 * x}$ |
| GERP score > 2 | Continuous | $0.622786748*\frac{1}{1+e^{-40 * (x-1.85)}}$ |
| In ultra-conserved elements | Discrete | 0.99974654 |
| In HOT regions | Discrete | 0.79722995 |
| In regulatory elements associated with genes | Discrete | 0.0028629 |
| Recurrent in multiple samples | Discrete | 1 |

**Dependency structure of features (leaf feature is a subset of root feature):**

In functional annotations

- In sensitive regions

- In ultra-sensitive regions

In functional annotations

- Motif-breaking score

In functional annotations

- In HOT regions

In regulatory regions associated with genes

- Network centrality score

In regulatory regions associated with genes

- Motif-gaining score

GERP score

- In ultra-conserved elements

**Table S3.** Weighted scoring scheme.

| Sample | GWAVA | CADD | FunSeq2 |
| --- | --- | --- | --- |
| MB67 - 931  (Medulloblastoma) | 17 | 86 | 2  7 (w/o recurrence) |
| MB59 - 2,183  (Medulloblastoma) | 25 | 224 | 2  13 (w/o recurrence) |
| HX9T - 8,183  (Liver cancer) | 125 | 898 | 31  60 (w/o recurrence) |
| HX10T - 9,259  (Liver cancer) | 134 | 1,036 | 21  67 (w/o recurrence) |
| HX11T - 8,432  (Liver cancer) | 136 | 929 | 34  82 (w/o recurrence) |
| HX13T - 21,507  (Liver cancer) | 363 | 2,297 | 87  203 (w/o recurrence) |
| HX17T - 20,909  (Liver cancer) | 400 | 2,632 | 77  195 (w/o recurrence) |

**Table S4.** Rankings of the *TERT* promoter mutation in seven cancer samples. ‘Matched region’ model is used for GWAVA (Figure S10).

|  | **Approximate time (min)** |
| --- | --- |
| GWAVA | 5 |
| CADD | 4-5 |
| FunSeq2 | 2 |

**Table S5.** Time comparisons using about 2,000 variants.

# Documentation

Our framework consists of two modules: building data context and variant prioritization.

## Building data context

We offer a flexible framework for users to incorporate their own data into the data context. All the data files used in the current data context can be replaced with user-specific data. Below is the detailed description. Scripts can be found under ‘Downloads’ of the web server.

* Define novel sensitive/ultra-sensitive regions

We provide scripts for users to define novel conserved regions in human populations. The algorithm is described in [1]. To define sensitive/ultra-sensitive regions, users need to prepare category files in BED format. The BED files contain the region coordinates under particular categories. For example, the BED file for category - ‘GATA1 binding sites’ - has all the binding coordinates of transcription factor GATA1. Scripts will identify categories under strong human-specific negative selection and define those categories as sensitive/ultra-sensitive regions based on the selection pressure. We use the criteria - enrichment of rare variants (depletion of common variants) - to measure negative selection constraints.

‘0.define.proximal.distal.regions.pl’. We provide this script for users to split categories into proximal or distal subsets. The proximal or distal subsets can be used as new categories.

Scripts used to identify sensitive/ultra-sensitive regions from scratch -‘1.Randomization.pl’ and ‘1.2.FDR.r’. ‘1.Randomization.pl’ uses GSC (genome structure correction) like method to generate null distributions for enrichment of rare variants for each category. ‘1.2.FDR.r’ calculates FDR using the randomization. This script can also be used to generate significant categories based on user-selected FDR.

Scripts used to identify novel sensitive/ultra-sensitive regions, in addition to those defined in [1] - ‘2.sensitive.regions.delta.increment.pl’. This script is only applicable to small number of categories (approximately 5).

Note: please prepare your polymorphisms file with only non-coding variants.

* Process GENCODE GTF file

We provide ‘3.gencode.process.pl’ to process GENCODE GTF file to obtain necessary files for data context. The script will generate ‘promoter’, ‘cds’, ‘intron’, and ‘UTR’ region files, which are used by the variant prioritization step. The ‘cds’ file could also be used to filter polymorphisms to obtain non-coding variants. Please put all the generated GENCODE files under ‘data/gencode’. GENCODE version 16 is used in the current data context.

* Add new networks

The networks used are under ‘data/networks’ folder. The tool will automatically read all the files in the folder and use the first field separated by ‘.’ as the network name. For example, ‘PPI.degree’ file will be used as network ‘PPI’. So to add new networks, simply put the network files into this folder and use the first field to denote the network name.

The files under the folder have two columns: ‘gene name’ and ‘centrality’. We provide ‘4.network.analysis.r’ for users to generate these files (either degree or betweenness centrality) from tab-delimited network files. Tab-delimited network files are two-column files showing the interacting genes (for each row, ‘gene A’ ‘gene B’).

* Identify potential target genes of regulatory elements

We have packaged our computer programs and current Roadmap Epigenomics Mapping Consortium (REMC) data as a software pipeline for users to define DRMs and identify their potential targets on their own data files. Scripts can be found under ‘Downloads’ of the web server. The scripts are written in C/C++. Please note that the data files are huge (about 40 G).

The pipeline involves the following three main steps:

a. Read user-defined regulatory regions, annotation file, tssEU expression, and meta-data of the data files (file names, total reads, and so on).

b. Calculate activity and inactivity levels at the DRMs based on the Roadmap Epigenomics data.

c. Correlate the activity/inactivity levels with the tssEU expression levels and determine their statistical significance, either using the pre-computed values or to compute the significance values on the fly based on the user-defined regulatory regions.

* Add new gene lists to annotate variants

The procedure is similar to ‘Add new networks’. Users can just put new files under ‘data/gene_lists’ folder and use the first field separated by ‘.’ as the gene list name.

* Add recurrent data for new cancer types

This is similar to ‘Add new networks’. Please put files under ‘data/cancer_recurrence’ and use the first field as the cancer type name. This file can be produced by running FunSeq2 (file ‘Recur.Summary’ produced by the tool) on cancer samples of a particular type.

* Add user-specific annotation sets, such as epigenetic modifications.

Please put files under directory ‘data/user_annotations’ or specific directory with option (-ua). The first field separated by ‘.’ will be used as annotation name. Please prepare your files in BED format and use the fourth column for additional information, if needed. We have placed repeat regions obtained from UCSC there as an example.

* All of other files can be replaced with user-specific data. Please refer to the files under ‘data/’ to correctly format them.

## Variant prioritization

1. Code structure

‘Funseq2/lib/Funseq_SNV.pm’ contains all subroutines used for SNVs analysis; ‘Funseq2/lib/Funseq_Indel.pm’ contains subroutines for indels analysis; ‘scripts/funseq2.pl’ stores the data path and organizes the subroutines into pipeline; ‘scripts/differential_gene_expression.r’ is an R script to detect differentially expressed genes between cancer and normal samples; ‘run.sh’ accepts the input parameters and passes them to ‘funseq2.pl’.

2. Dependencies

The proper execution of the tool depends on the following tools.

* sed, awk, grep

* bedtools *(version bedtools-2.17.0) (*[*http://code.google.com/p/bedtools/downloads/list*](http://code.google.com/p/bedtools/downloads/list)*)*

*For intersection analysis and sequence retrieval.*

* tabix *(version tabix-0.2.6 and up) (*[*http://sourceforge.net/projects/samtools/files/tabix/*](http://sourceforge.net/projects/samtools/files/tabix/)*)*

* VAT (variant annotation tool - snpMapper, indelMapper Modules) *(*[*http://vat.gersteinlab.org/index.php*](http://vat.gersteinlab.org/index.php)*)*

*If you are only interested in non-coding variants, you don't need to install VAT. But remember to use '-nc' option.*

* TFMpvalue-sc2pv *(*[*http://bioinfo.lifl.fr/TFM/TFMpvalue/*](http://bioinfo.lifl.fr/TFM/TFMpvalue/)*)*

*Calculate P values of sequence scores with respect to PWMs.*

* bigWigAverageOverBed *(*[*http://hgdownload.cse.ucsc.edu/admin/exe/linux.x86_64/*](http://hgdownload.cse.ucsc.edu/admin/exe/linux.x86_64/)*)*

*Retrieve GERP scores. Note that GERP data file is approximately 7 G. If you are not interested in GERP scores, the GERP file and bigWigAverageOverBed are not needed.*

* R *(*[*http://www.r-project.org*](http://www.r-project.org)*)*

*Only needed for differential gene expression analysis.*

* Perl package Parallel::ForkManager *(*[*http://search.cpan.org/~szabgab/Parallel-ForkManager-1.03/lib/Parallel/ForkManager.pm*](http://search.cpan.org/~szabgab/Parallel-ForkManager-1.03/lib/Parallel/ForkManager.pm)*)*

*Required for parallel running.*

*Please make sure you have Perl 5 and up.*

3. Tool installation

This is a PERL- and Linux/UNIX-based tool. At the command-line prompt, type the following. The purpose is to write the path of perl modules to the environment.

*$ tar xvf funseq2.1.0.tar*

*$ cd funseq2-1.0/*

*$ cd Funseq2/*

*$ perl Makefile.PL*

*$ make*

*$ make install*

If you don’t have the permission to modify the environment, open the ‘.bashrc’ file and add the following to the end of the file. Then ‘*source .bashrc’*.

*PERL5LIB=${PERL5LIB}: $path_of_the_tool/funseq2-1.0/Funseq2/lib*

*export PERL5LIB*

4. Pre-built data context

All of the data can be downloaded under ‘Downloads’ in the web server. If you would like to use the data, please download and put them under ‘*funseq2-1.0/data*’.

5. Running the tool

To display the usage of tool, type ‘.*/run.sh*’.

_______________________________________________________________________

** Usage: ./run.sh -f file -maf MAF -m <1/2> -inf <bed/vcf> -outf <bed/vcf> -nc -o path -g file -exp file -cls file -exf <rpkm/raw> -p int -cancer cancer_type -s score -uw -ua user_annotations_directory*

*Options :*

*-f [Required] User Input SNVs File*

*-inf [Required] Input format - BED or VCF*

*-maf [Optional] Minor Allele Frequency Threshold to filter 1KG SNVs,default = 0*

*-m [Optional] 1 - Somatic Genome (default); 2 - Germline or Personal Genome*

*-outf [Optional] Output format - BED or VCF,default is VCF*

*-nc [Optional] Only do non-coding analysis, no need of VAT (variant annotation tool)*

*-o [Optional] Output path, default is the directory 'out'*

*-g [Optional] gene list, only output variants associated with selected genes.*

*-exp [Optional] gene expression matrix*

*-cls [Optional] class file for samples in gene expression matrix*

*-exf [Optional] gene expression format - rpkm / raw*

*-p [Optional] Number of genomes to parallel, default = 5*

*-cancer [Optional] cancer type from recurrence database, default is all of the cancer type*

*-uw [Optional] Use unweighted scoring scheme, defalut is weighted*

*-s [Optional] Score threshold to call non-coding candidates, default = 1.5 for weighted scoring & default = 5 for unweighted scoring*

*-ua [Optional] Directory containing user annotations. Default is to read from ‘data/user_annotations’.*

*-db [Optional] Use the recurrence database to score variants. Recurrence gets a additional score.*

** Multiple Genomes with Recurrent Output*

*Option 1: Separate multiple files by ','*

*Example: ./run.sh -f file1,file2,file3,... -maf MAF -m <1/2> -inf <bed/vcf> -outf <bed/vcf> ...*

*Option 2: Use the 6th column of BED file to specify samples*

*Example: ./run.sh -f file -maf MAF -m <1/2> -inf bed -outf <bed/vcf> ...*

*NOTE: Please make sure you have sufficient memory, at least 3G.*

_________________________________________________________________

-maf : should be a number between 0~1

-nc : when using this option, users don’t need to install VAT (variant annotation tool)

-exp, -cls, -exf : if used, should be specified together.

-m : We also provide the option for germline or personal genomes, which compare mutated allele with ancestral allele, since the functional impact of variants reflects the historical event when the polymorphism was first introduced in the human populations.

6. Input file format

* User input file (-f): could be either BED or VCF format. For indels, please use “-” instead of other symbols in ‘allele’ columns for insertions or deletions. Indels will be analyzed for BED format.

BED format. In addition to the three required BED fields, please prepare your files as following (five required fields, tab delimited; **the sixth column is reserved for sample names, do not put other information there**): chromosome, start position, end position, reference allele, and alternative allele.

Chromosome - name of the chromosome (for example, chr3, chrX)

Start position - start coordinates of variants. (0-based)

End position - end coordinates of variants. (end exclusive)

for example, chr1 0 100 spanning bases numbered 0-99

Reference allele - germline allele of variants

Alternative allele - mutated allele of variants

VCF format. The header line names the eight fixed, mandatory columns. These columns are as follows (tab-delimited):

 #CHROM POS ID REF ALT QUAL FILTER INFO

Recurrent analysis input format

Option 1: separated files for each genome (BED or VCF). Use *“-f file1, file2, file3*” separated by comma.

Option 2: put all variants in one file (only for BED format, use the sixth column labeling sample names). Use “*-f file*”.

* Gene list format (-g): If you are interested in particular set of genes, you can put your genes in one file (one gene per row) and use “*-g file*” to only analyze variants in or associated with those genes. Please use **gene symbols**.

* Gene expression format (-exp): Users can also upload gene expression file for the program to detect differentially expressed genes between cancer and benign samples and highlight variants associated with these genes. The gene expression file should be prepared as a matrix with first column stores gene names (use **gene symbols**) and first row as sample names. Other fields are gene expression data either in RPKM or raw read counts format. Tab delimited.

For example,

| *Gene* | *Sample1* | *Sample2* | *Sample3* | *Sample4* | *…* |
| --- | --- | --- | --- | --- | --- |
| *A1BG* | *1* | *5* | *40* | *0* | *…* |
| *A1CF* | *20* | *9* | *0* | *23* | *…* |
| *…* | *…* | *…* | *…* | *…* | *…* |

* Sample class format (-cls): In addition to the expression file, users need to upload a file with samples annotated as ‘cancer’ or ‘benign’ (only two classes ‘**cancer**’ or ‘**benign**’). The number of samples in this file should be equal to that in expression data. And sample names should match.

For example,

| *Sample1* | *Benign* |
| --- | --- |
| *Sample2* | *Cancer* |
| *Sample3* | *Cancer* |
| *Sample4* | *Benign* |
| *…* | *…* |

7. Output files

Five output files will be generated: ‘*Output.format*’, ‘*Output.indel.format*’,‘*Recur.Summary*’, ‘*Candidates.Summary*’, and ‘*Error.log*’. *Output.format:* stores detailed results for all samples; *Output.indel.format:* contains results for indels; *Recur.Summary:* the recurrence result when having multiple samples; *Candidates.Summary:* brief output of potential candidates (coding non-synonymous/premature-stop variants, non-coding variants with score (>= 5 for un-weighted scoring scheme and >=1.5 for weighted scoring scheme) and variants in or associated with known cancer genes); *Error.log:* error information. For un-weighted scoring scheme, each feature is given value 1.

When provided with gene expression files, two additional files will be produced - ‘*DE.gene.txt*’ stores differentially expressed genes and ‘*DE.pdf* ’is the differential gene expression plot.

* Sample BED format output

Header:

*chr start end ref alt sample gerp;cds;variant.annotation.cds;network.hub;gene.under.negative.selection;ENCODE.annotated;hot.region;motif.analysis;sensitive;ultra.sensitive;ultra.conserved;target.gene[known_cancer_gene/TF_regulating_known_cancer_gene,differential_expressed_in_cancer,actionable_gene];coding.score;noncoding.score;recurrence.within.samples;recurrence.database*

Coding variant:

*chr1 36205041 36205042 C A PR2832 5.6;Yes;VA=1:CLSPN:ENSG00000092853.9:-:prematureStop:4/4:CLSPN-001:ENST00000251195.5:3999_3232_1078_E->*:CLSPN-005:ENST00000318121.3:4020_3232_1078_E->*:CLSPN-003:ENST00000373220.3:3828_3040_1014_E->*:CLSPN-004:ENST00000520551.1:3861_3073_1025_E->*;PPI;Yes;.;.;.;.;.;.;CLSPN;5;.;.;.*

Non-coding variant:

*chr6 152304995 152304996 A G PR2832 2.63;No;.;ESR1:PHOS(0.276)PPI(0.995)REG(0.994);.;.;.;.;.;.;.;ESR1(Intron)[TF_regulating_known_cancer_gene:H3F3A,MN1,PRCC,RARA,SLC34A2,TPM3][actionable];.;1.60983633568013;.;.*

* Sample VCF format output

Header:

*##fileformat=VCFv4.0*

*##INFO=<ID=OTHER,Number=.,Type=String, Description = "Other Information From Original File">*

*##INFO=<ID=SAMPLE,Number=.,Type=String,Description="Sample id">*

*##INFO=<ID=CDS,Number=.,Type=String,Description="Coding Variants or not">*

*##INFO=<ID=VA,Number=.,Type=String,Description="Coding Variant Annotation">*

*##INFO=<ID=HUB,Number=.,Type=String,Description="Network Hubs, PPI (protein protein interaction network), REG (regulatory network), PHOS (phosphorylation network)...">*

*##INFO=<ID=GNEG,Number=.,Type=String,Description="Gene Under Negative Selection">*

*##INFO=<ID=GERP,Number=.,Type=String,Description="Gerp Score">*

*##INFO=<ID=NCENC,Number=.,Type=String,Description="NonCoding ENCODE Annotation">*

*##INFO=<ID=HOT,Number=.,Type=String,Description="Highly Occupied Target Region">*

*##INFO=<ID=MOTIFBR,Number=.,Type=String,Description="Motif Breaking">*

*##INFO=<ID=MOTIFG,Number=.,Type=String,Description="Motif Gain">*

*##INFO=<ID=SEN,Number=.,Type=String,Description="In Sensitive Region">*

*##INFO=<ID=USEN,Number=.,Type=String,Description="In Ultra-Sensitive Region">*

*##INFO=<ID=UCONS,Number=.,Type=String,Description="In Ultra-Conserved Region">*

*##INFO=<ID=GENE,Number=.,Type=String,Description="Target Gene (For coding - directly affected genes ; For non-coding - promoter or distal regulatory module)">*

*##INFO=<ID=CANG,Number=.,Type=String,Description="Prior Gene Information, e.g.[cancer][TF_regulating_known_cancer_gene][up_regulated][actionable]...";*

*##INFO=<ID=CDSS,Number=.,Type=String,Description="Coding Score">*

*##INFO=<ID=NCDS,Number=.,Type=String,Description="NonCoding Score">*

*##INFO=<ID=RECUR,Number=.,Type=String,Description="Recurrent elements / variants">*

*##INFO=<ID=DBRECUR,Number=.,Type=String,Description="Recurrence database">*

*#CHROM POS ID REF ALT QUAL FILTER INFO*

Coding variant:

*chr1 36205042 . C A . . SAMPLE=PR2832;GERP=5.6;CDS=Yes;VA=1:CLSPN:ENSG00000092853.9:-:prematureStop:4/4:CLSPN-001:ENST00000251195.5:3999_3232_1078_E->*:CLSPN-005:ENST00000318121.3:4020_3232_1078_E->*:CLSPN-003:ENST00000373220.3:3828_3040_1014_E->*:CLSPN-004:ENST00000520551.1:3861_3073_1025_E->*;HUB=PPI;GNEG=Yes;GENE=CLSPN;CDSS=5*

Non-coding variant:

*chr6 152304996 . A G . . SAMPLE=PR2832;GERP=2.63;CDS=No;HUB=ESR1:PHOS(0.276)PPI(0.995)REG(0.994);GENE=ESR1(Intron);CANG=ESR1[TF_regulating_known_cancer_gene:H3F3A,MN1,PRCC,RARA,SLC34A2,TPM3][actionable];NCDS=1.60983633568013*

***** Output description (VCF format as an example)

#### VA (variants annotation)

This is the output produced from VAT (variant annotation tool) for coding variations. Please refer to ‘<http://vat.gersteinlab.org>’ for documentations.

####

#### NCENC (Non-coding ENCODE annotation)

Example: *‘NCENC=TFP(CEBPB|chr5:139639150-139639496),TFP(STAT3|chr5:139638936-139640136),TFP(STAT3|chr5:139638976-139639553),TFP(STAT3|chr5:139638989-139639544),TFP(STAT3|chr5:139638999-139639716)’*

This is formatted as “*category(element_name|chromosome:coordinates)*” (0-based, end exclusive).

*TFP - transcription factor binding peak.*

*TFM - transcription factor bound motifs in peak regions.*

*DHS - DNase1 hypersensitive sites, with number of cell lines (MCV, total 125 cell lines).*

*ncRNA - non-coding RNA*

*Pseudogene*

*Enhancer - chmm/segway (genome segmentation), drm (distal regulatory module)*

####

#### HOT (transcription factor highly occupied region)

Example: *‘HOT=Helas3’*

If a variant occurs in HOT regions, the corresponding cell lines (five in total) are shown. This annotation is from [2].

#### MOTIFBR (motif-breaking analysis)

SNV Example: *‘MOTIFBR=MAX#Myc_known9_8mer#102248644#102248656#-#9#0.068966#0.931034’*

The variant causes a motif-breaking event. This field is a hash tag delimited, defined as follows:  *TF name # motif name # motif start # motif end # motif strand # mutation position # alternative allele frequency in PWM # germline allele frequency in PWM* . (0-based, end exclusive)

Indel Example: *‘MOTIFBR=TCF12#TCF12_disc5_8mer#115719379#115719390#+’*

This field is a hash tag delimited, defined as follows:  *TF name # motif name # motif start # motif end # motif strand.* (0-based, end exclusive)

####

#### MOTIFG (motif-gaining analysis)

SNV example: *‘MOTIFG=GATA_known5#75658824#75658829#-#1#4.839#4.181’*

The variant causes a motif-gaining event. Hash tag delimited: *motif name # motif start # motif end # motif strand # mutation position # sequence score with alternative allele # sequence score with germline allele*. (0-based, end exclusive)

Indel example: *‘MOTIFG=Ets_known10#CGGAAA#6#+#5.743’*

Hash tag delimited: *motif name # motif sequence discovered # motif length # motif strand # sequence score with alternative allele*.

*GENE (target gene - for coding: directly affected genes; for non-coding: promoter or distal regulatory module)*

Example: *‘GENE=ARNT2(Distal),C15orf26(Intron),IL16(Distal)’*

#### For non-coding variants, ‘intron’, ‘promoter’, ‘UTR’, ‘Distal’ and ‘Medial’ tags are annotated. For ‘Distal’ and ‘Medial’ tags, the corresponding association score (with histone modifications) is also shown. ‘Distal’ means that the regulatory element is >10 kb away from TSS, whereas ‘Medial’ means within 10 kb.

#### CANG (cancer related information)

Example: *‘CANG=EGFR[actionable][cancer]’*

This field stores all gene related information. Currently there are five possible tags:

*[cancer]: the gene have been annotated as an cancer gene.*

*[TF_regulating_known_cancer_gene]: the gene is a transcription factor regulating known cancer genes. The regulated cancer genes are also shown.*

*[actionable]: the gene is potentially actionable (“druggable”).*

*[up_regulated]: the gene is upregulated in cancers, when providing RNA-Seq gene expression data.*

*[down_regulated]: the gene is downregulated in cancers, when providing RNA-Seq gene expression data.*

#### When user provides new gene lists, tags about these gene lists will be shown in this field.

#### USER_ANNO (user annotations)

Example: *‘USER_ANNO=REPEAT(FLAM_A|chr1:100544744-100544854)’*

This field stores all user provided annotations.

#### RECUR (recurrent genes, regulatory elements and mutations within samples)

Example: *‘RECUR=Pseudogene(ENST00000467115.1|chr1:568914-569121):PR1783(chr1:568941,chr1:569004*),PR2832(chr1:569004*)’*

When analyzing multiple samples, if genes or regulatory elements are shown in >= 2 samples, they are annotated as ‘*gene/regulatory element name: recurrent samples (variants in corresponding samples (position is 1-based))*’. If it is a same-site mutation, ‘*’ is tagged.

*DBRECUR (Recurrence databse)*

Example: *‘DBRECUR=Enhancer(chmm/segway|chr15:22517400-22521103):Lung_Adeno(Altered in 4/24(16.67%) samples.)|Prostate(Altered in 2/64(3.12%) samples.),Enhancer(drm|chr15:22517700-22521100):Lung_Adeno(Altered in 4/24(16.67%) samples.)|Prostate(Altered in 2/64(3.12%) samples.)’*

If genes, regulatory elements or mutations are observed in the recurrence database (currently including 570 samples of 10 cancer types and COSMIC), the recurrence information is shown here. ‘*recurrent element(name|coordinates):cancer type(recurrence information in this cancer type)*’. Recurrence information is separated by ‘,’.

# Web server

FunSeq2 is also implemented as a web server using Django web framework. Users can download the results or view them in interactive tables.

# References

1. Khurana E, Fu Y, Colonna V, Mu XJ, Kang HM, Lappalainen T, Sboner A, Lochovsky L, Chen J, Harmanci A, Das J, Abyzov A, Balasubramanian S, Beal K, Chakravarty D, Challis D, Chen Y, Clarke D, Clarke L, Cunningham F, Evani US, Flicek P, Fragoza R, Garrison E, Gibbs R, Gumus ZH, Herrero J, Kitabayashi N, Kong Y, Lage K, et al: **Integrative Annotation of Variants from 1092 Humans: Application to Cancer Genomics.** *Science* 2013, **342:**1235587.

2. Yip KY, Cheng C, Bhardwaj N, Brown JB, Leng J, Kundaje A, Rozowsky J, Birney E, Bickel P, Snyder M, Gerstein M: **Classification of human genomic regions based on experimentally determined binding sites of more than 100 transcription-related factors.** *Genome Biol* 2012, **13:**R48.
